# Single-cell DNA sequencing reveals a high incidence of chromosomal abnormalities in human blastocysts

**DOI:** 10.1172/JCI174483

**Published:** 2024-01-04

**Authors:** Effrosyni A. Chavli, Sjoerd J. Klaasen, Diane Van Opstal, Joop S.E. Laven, Geert J.P.L. Kops, Esther B. Baart

**Affiliations:** 1 Division of Reproductive Endocrinology and Infertility, Department of Obstetrics and Gynecology, Erasmus MC, University Medical Center Rotterdam, Rotterdam, Netherlands.; 2 Hubrecht Institute-KNAW (Royal Academy of Arts and Sciences) and University Medical Center Utrecht, Utrecht, Netherlands.; 3 Oncode Institute, Utrecht, Netherlands.; 4 Department of Clinical Genetics and; 5 Department of Developmental Biology, Erasmus MC, University Medical Center Rotterdam, Rotterdam, Netherlands.

**Keywords:** Genetics, Reproductive biology, Embryonic development, Fertility, Genetic instability

## Abstract

Aneuploidy, a deviation from the normal chromosome copy number, is common in human embryos and is considered a primary cause of implantation failure and early pregnancy loss. Meiotic errors lead to uniformly abnormal karyotypes, while mitotic errors lead to chromosomal mosaicism: the presence of cells with at least 2 different karyotypes within an embryo. Knowledge about mosaicism in blastocysts mainly derives from bulk DNA sequencing (DNA-Seq) of multicellular trophectoderm (TE) and/or inner cell mass (ICM) samples. However, this can only detect an average net gain or loss of DNA above a detection threshold of 20%–30%. To accurately assess mosaicism, we separated the TE and ICM of 55 good-quality surplus blastocysts and successfully applied single-cell whole-genome sequencing (scKaryo-Seq) on 1,057 cells. Mosaicism involving numerical and structural chromosome abnormalities was detected in 82% of the embryos, in which most abnormalities affected less than 20% of the cells. Structural abnormalities, potentially caused by replication stress and DNA damage, were observed in 69% of the embryos. In conclusion, our findings indicated that mosaicism was prevalent in good-quality blastocysts, whereas these blastocysts would likely be identified as normal with current bulk DNA-Seq techniques used for preimplantation genetic testing for aneuploidy.

## Abstract 2

Single-cell karyotype sequencing reveals chromosomal mosaicism to be common in trophectoderm and inner cell mass of human blastocysts, and provides insight into aneuploidy driving mechanisms.

## Graphical abstract

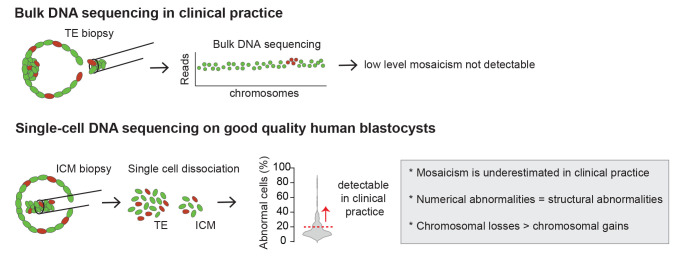

## Introduction

Human preimplantation embryos are prone to chromosomal instability, resulting in the gain or loss of chromosomal content (1). As this is detrimental to the fitness of embryonic cells, it is considered to be the major cause of miscarriages and congenital birth defects (2). Aneuploidy can occur as a numerical chromosomal abnormality, involving the gain or loss of a whole chromosome, whereas partial aneuploidy involves a structural abnormality, in which only a chromosomal segment is affected. These abnormalities may derive either from meiotic errors that result in gametes with an abnormal chromosomal set, or from mitotic errors that occur postzygotically (3, 4). Meiotic errors lead to fully abnormal embryos containing cells with the same abnormality. Mitotic errors affect only part of the cells, leading to 2 or more distinct cytogenetic cell populations within an embryo, defined as chromosomal mosaicism. Normal and (partial) aneuploid cell populations lead to diploid-aneuploid mosaicism, whereas different (partial) aneuploid cell populations lead to aneuploid mosaicism (1, 5).

Mosaicism is proposed to be the result of the error-prone nature of the first few mitotic cleavage divisions (1, 6). Reported rates of chromosomal mosaicism in cleavage-stage embryos vary between 15% and 91% (4, 6–10). The incidence of both uniform aneuploidy and mosaicism appears to decline as embryo development progresses toward the blastocyst stage (11, 12), likely due to developmental arrest of aneuploid embryos and/or selective loss of aneuploid cells (12, 13). Knowledge about chromosomal mosaicism at the blastocyst stage is mainly based on cytogenetic analysis of multicellular trophectoderm (TE) and/or inner cell mass (ICM) samples with next-generation sequencing (NGS), which has been shown to be the most sensitive technique to identify numerical and structural abnormalities (14). Using this method, the reported incidence of mosaicism for numerical abnormalities ranges between 14% and 59% for good-quality blastocysts (15–18). Structural abnormalities were shown to be mostly of mitotic origin and were reported in 2.4%–31% of blastocysts (10, 19–21). However, in the most commonly used NGS platforms, abnormalities are detectable when present in at least 20%–30% of the analyzed cells (22), and consequently, low-level mosaicism will go undetected (23). Moreover, since NGS is performed on bulk DNA, it will not detect cells with reciprocal chromosome abnormalities (i.e., a mitotic event in which 1 daughter cell ends up with a trisomy and the other with the reciprocal monosomy), if there is no net average chromosome gain or loss (23). As a result, the true incidence of mosaicism at the blastocyst stage is still unknown, and its consequences for embryo selection during preimplantation genetic testing for aneuploidy (PGT-A) are extensively debated (15, 24–26).

Although laborious single-cell methods such as FISH have been used to investigate chromosomal mosaicism in human blastocysts (4, 7, 11), comprehensive chromosome screening on a single-cell level for all cells can give deeper insights because all chromosomes and subchromosomal regions can be examined (27). Two such approaches have been described but are limited by error-prone methods based on single-cell RNA-Seq or by using only a limited number of cells per embryo (27–29). Comprehensive single-cell chromosome analysis could also reveal important information regarding underlying mechanisms. For instance, it is hypothesized that aneuploid cells in the 2 embryonic lineages, TE and ICM, behave differently, leading to preferential allocation of aneuploid cells in the TE but not the ICM (30, 31).

To investigate the chromosomal content of single-cells from human blastocysts donated for research, we applied single-cell whole-genome sequencing (scKaryo-Seq) (32, 33), which allowed us to study both numerical and structural abnormalities with high accuracy. We distinguished meiotic from mitotic errors and speculated on mitotic error events contributing to chromosomal mosaicism. TE and ICM were analyzed separately in order to explore whether there was an overrepresentation of abnormal cells in either lineage, which also enabled us to investigate the developmental timing of the mitotic error events.

## Results

### scKaryo-Seq accurately detects chromosomal abnormalities in control cells and human blastocysts.

scKaryo-Seq has previously been shown to allow for high-fidelity determination of the copy number state of all chromosomes in single cells in a high-throughput setting (33). We first confirmed that scKaryo-Seq also would detect known numerical and structural abnormalities in manually plated cells, observing an accuracy of 99.5% (Supplemental Methods). We detected unexpected additional abnormalities in 28 of 376 (7.4%) control cells. To assess whether these additional abnormalities had a biological rather than a technical origin, we examined whether these cells undergo occasional errors in chromosome segregation. We indeed observed abnormal anaphases and micronuclei, a widely used marker of chromosomal instability, in a similar proportion of the control cells (Supplemental Figure 1 and Supplemental Methods; supplemental material available online with this article; https://doi.org/10.1172/JCI174483DS1).

**Figure 1:**
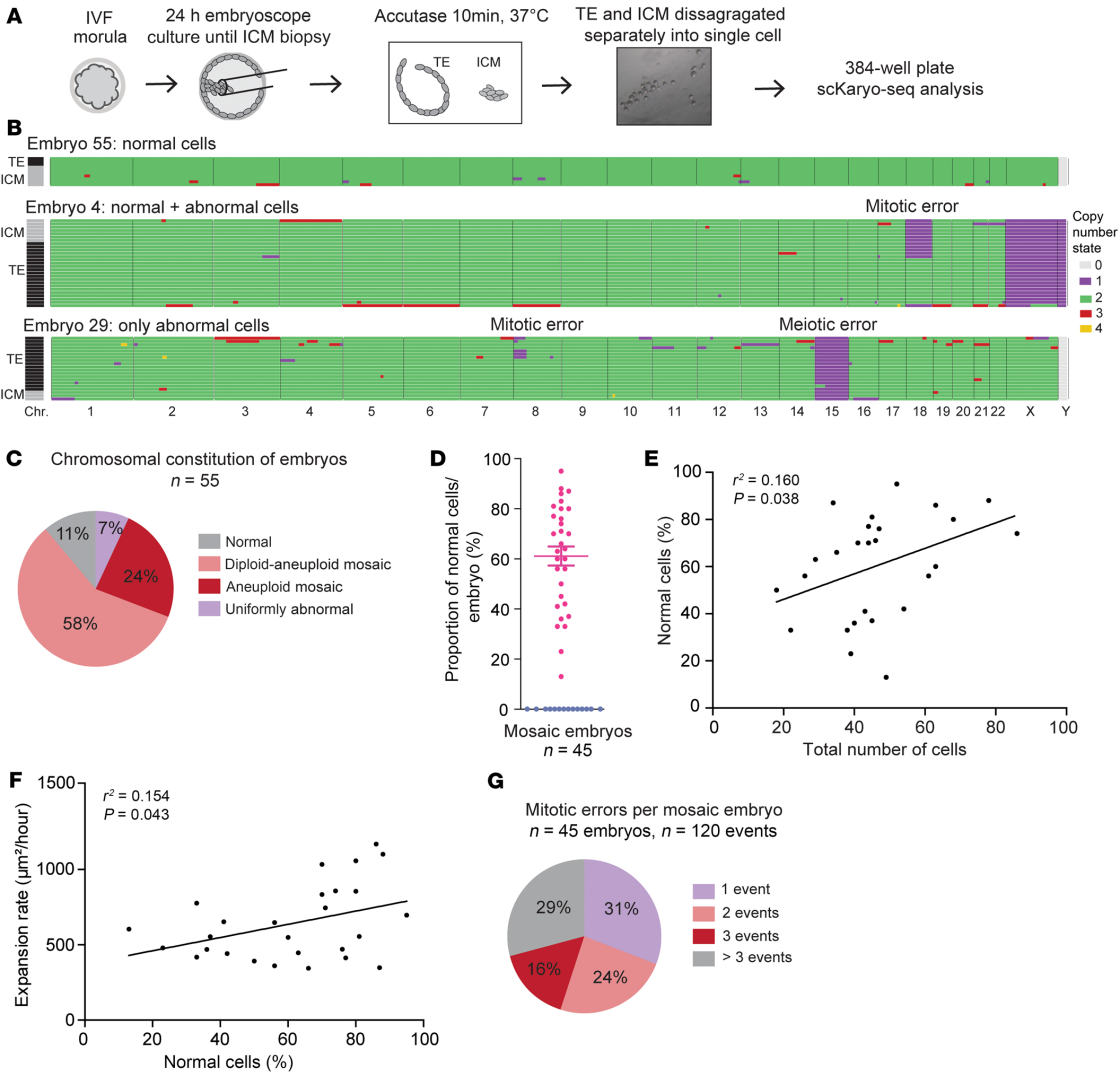
Chromosomal mosaicism is common in human blastocysts. (**A**) Schematic depicting the embryo biopsy and disaggregation procedure for scKaryo-Seq. (**B**) Examples of scKaryo-Seq results as genome-wide copy number plots of 3 embryos, which had either normal cells (embryo 55, *n* = 10 cells), normal and abnormal cells (embryo 4, *n* = 27 cells), or only abnormal cells (embryo 29, *n* = 20 cells). Embryo 4 and embryo 29 are both mosaic, as mitotic errors are involved. Every row represents a single cell and every column is a different chromosome. The colors portray copy number states. All abnormalities are presented regardless of the quality control result. Colors on the left depict TE (black) or ICM cells (gray). The embryo numbers refer to Supplemental Table 3. For these embryos, embryoscope videos are available showing normal morphological development (Supplemental Videos 1–3). (**C**) Pie chart of the percentage of embryos that had only normal cells (normal, *n* = 6 of 55), normal and abnormal cells (diploid-aneuploid mosaic, *n* = 32 of 55), cytogenetically different abnormal cells (aneuploid mosaic, *n* = 13 of 55), and cytogenetically identical abnormal cells (uniformly abnormal, *n* = 4 of 55). (**D**) Percentage of normal cells per mosaic embryo. Diploid-aneuploid and aneuploid mosaic embryos are depicted in pink and purple, respectively (data indicate the mean ± SEM.) (**E**) Correlation between the percentage of chromosomally normal cells and the total number of disaggregated cells per embryo (*n* = 27, linear regression). (**F**) Correlation between the percentage of chromosomally normal cells and the blastocyst expansion rate per embryo (*n* = 27, linear regression). (**G**) Pie chart of the percentage of mosaic embryos affected by 1, 2, 3, or more than 3 events (mitotic error). Common abnormalities within daughter cells are considered to be the result of 1 event.

Next, we aimed to investigate human blastocysts. We successfully thawed 129 morula-stage embryos, from which 79 developed into good-quality blastocysts with a clearly discernible ICM that allowed biopsy (Figure 1A). We used time-lapse imaging to monitor this development and performed embryo disaggregation within 9–32 hours from the start of blastulation. Twenty-four embryos were excluded from the analysis, as both biopsy and single-cell distribution in plates failed or the sequencing results were inconclusive. From the remaining 55 embryos, the TE and ICM were successfully separated for 52 embryos. After sequencing and quality control, a successful cytogenetic result of at least 1 cell per embryonic lineage was obtained for 41 embryos. For 11 embryos, only cells from the TE (*n* = 9) or ICM (*n* = 2) were successfully sequenced. Three embryos were not biopsied and were disaggregated as a whole. In total 1,057 of 2,322 cells were successfully analyzed (45%), 535 of which had an abnormal chromosomal content. Per embryo, on average, 42% of isolated cells were successfully karyotyped (Supplemental Table 2). The cytogenetic results per embryo and per cell lineage are shown as genome-wide copy number plots (Figure 1B, Supplemental Table 3, and Supplemental Figure 2).

### Most human blastocyst–stage embryos are mosaic.

A low percentage of embryos showed an identical chromosomal constitution in all cells: 11% contained only normal cells, and 7% had the same abnormal chromosome constitution, indicative of a meiotic origin (Figure 1C). Chromosomal mosaicism was observed in 82% of embryos. This group contained embryos with diploid-aneuploid mosaicism (58%), in which, on average, 60% of cells were normal (Figure 1D), as well as embryos with aneuploid mosaicism (24%). Aneuploid mosaic embryos contained cells with at least 1 abnormality in common, indicating a meiotic origin, whereas part of these cells had additional mitotic abnormalities. Exceptions to this were 3 embryos containing different abnormal cells with genome-wide anomalies (embryos 42, 43, and 44; Supplemental Table 3). To investigate the biological importance of mosaicism for developmental potential in the diploid-aneuploid mosaic embryos, we investigated the correlation between the proportion of chromosomally normal cells observed within the embryo and developmental characteristics. We found a significant correlation between the proportion of normal cells and both the total number of cells (Figure 1E), as well as the blastocyst expansion rate (Figure 1F), a parameter previously shown to correlate with both implantation potential (34) and aneuploidy (35). This strongly suggests that the sample of the cells we were able to successfully analyze per embryo reflected the chromosomal constitution of the whole embryo.

In 69% of the mosaic embryos, more than 1 mitotic error event was involved (Figure 1G). In most of the mosaic embryos, we observed the same or reciprocal chromosomal abnormality(ies) in more than 1 cell, making it unlikely that these abnormalities were the result of a technical artifact. In only 7 of 45 mosaic embryos, the observed abnormalities were restricted to single cells, but ones with high-quality scKaryo-Seq profiles (Supplemental Table 3). Hence, we conclude that the majority of the good-quality human blastocysts we studied here were mosaic.

### Distribution of abnormal cells between TE and ICM and developmental timing of mitotic errors.

**Figure 2:**
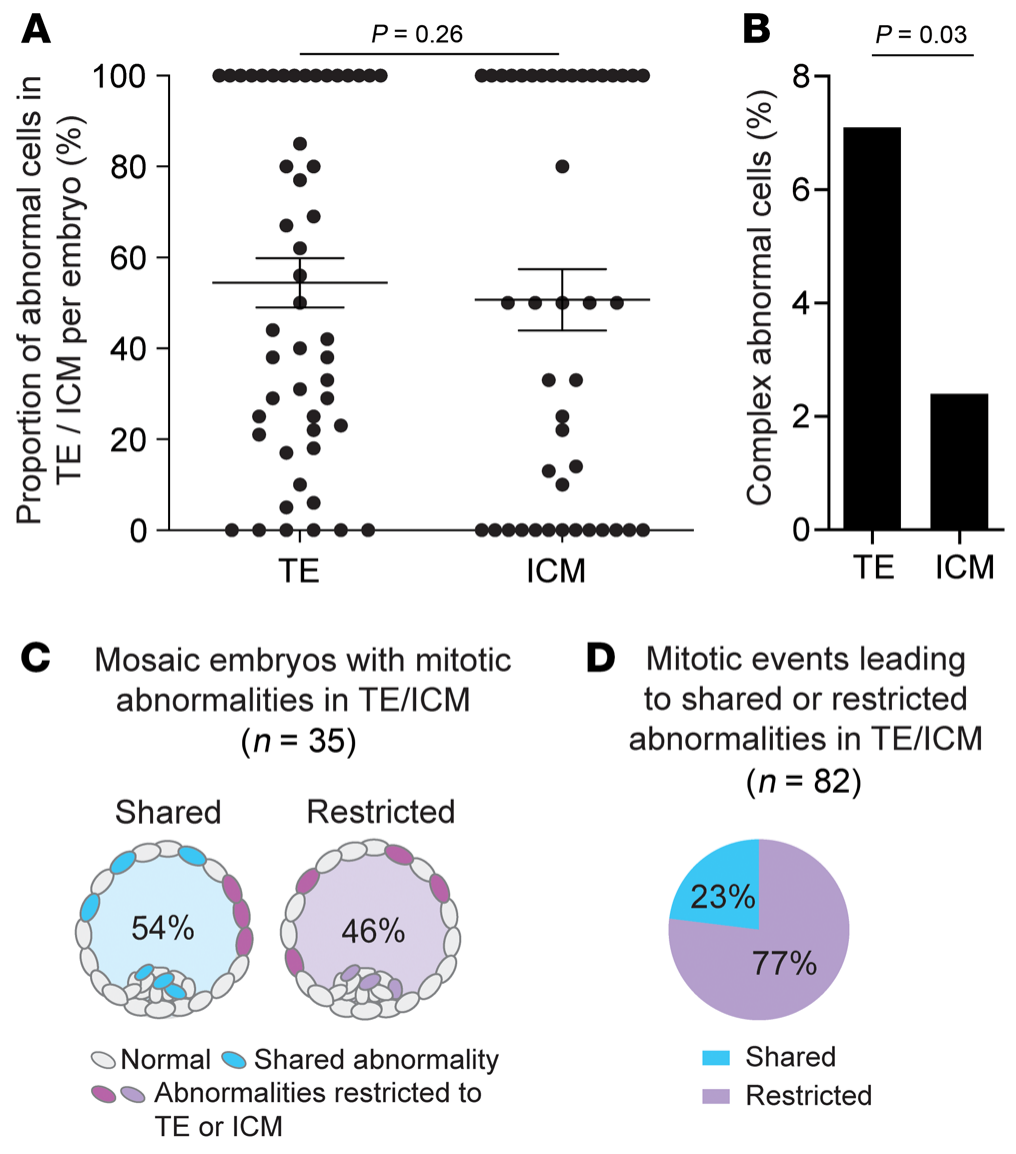
Chromosomal abnormalities in the TE versus the ICM. (**A**) Proportion of abnormal cells in the TE versus the ICM per embryo (*n* = 52). Data indicate the mean ± SEM. *P* = 0.26, by Fisher’s exact test. Embryos in which the TE and ICM were not separated are excluded. (**B**) Percentage of complex abnormal cells in the TE (*n* = 58 of 812) versus the ICM (*n* = 4 of 160). *P* = 0.03, by Fisher’s exact test. Complex cells have more than 4 chromosomal abnormalities. (**C**) Percentage of embryos that shared a mitotic abnormality in the embryonic lineages and percentage of embryos that had only abnormalities restricted to either the ICM or the TE. (**D**) Percentage of mitotic events that led to shared or restricted mitotic abnormalities in the embryonic lineages.

When analyzing the frequency of abnormal cells in relation to embryonic lineage, we found no evidence of preferential allocation of abnormal cells to either TE or ICM (Figure 2A). However, complex abnormal cells were more common in the TE (Figure 2B). In mosaic embryos with scKaryo-Seq results from both TEs and ICMs (*n* = 35), we examined mitotic abnormalities that were shared between the 2 embryonic lineages or restricted to either the TE or ICM to gain insight into the timing of the mitotic error event (Figure 2C). In 46% (*n* = 16 of 35) of embryos, there were no shared abnormalities between the 2 lineages, indicating that the mitotic error(s) likely occurred after embryonic lineage specification, or that the daughter cells ended up within 1 lineage. In 54% (*n* = 19 of 35) of mosaic embryos, the ICM and TE shared at least 1 chromosomal abnormality, indicating that this mitotic error took place before cell lineage specification. However, these embryos also had other abnormalities that were restricted to one of the lineages and possibly occurred after embryonic lineage specification. Assuming that abnormalities affecting the same chromosome (segment) in daughter cells originated from 1 error event, we were able to detect 82 mitotic events in our data set. From these events, 23% (*n* = 19 of 82) probably occurred before lineage specification, whereas 77% (*n* = 63 of 82) occurred after lineage specification (Figure 2D). Although it is possible that the products of a mitotic error event prior to lineage specification could end up in only 1 lineage, it is unlikely that this occurred in the majority of mitotic error events.

### TE mosaicism is underestimated when using bulk DNA sequencing.

**Figure 3:**
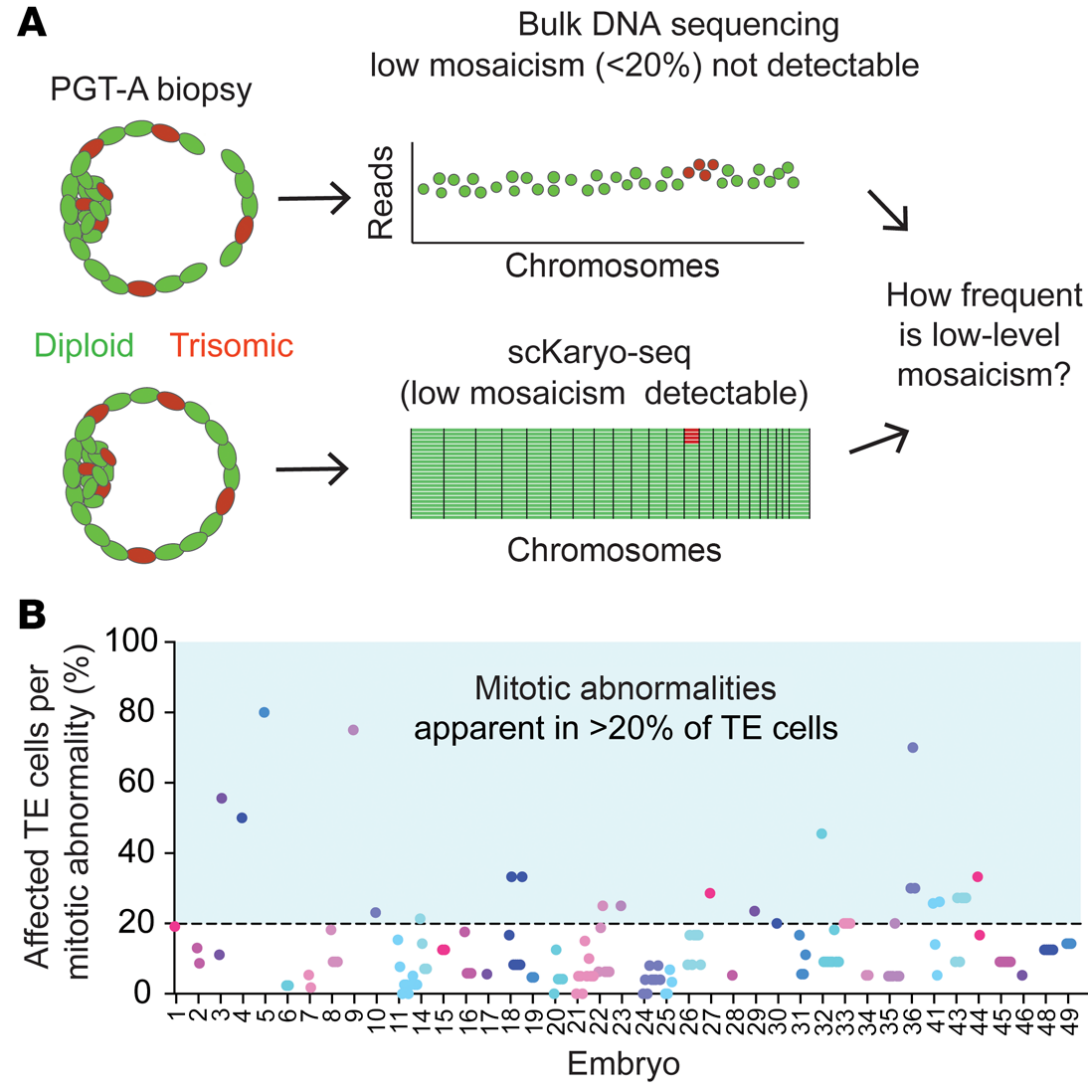
Mosaicism is underestimated with bulk DNA-Seq methods. (**A**) Schematic depicting the experimental approach to determine whether single-cell sequencing improves the detection of chromosomal mosaicism compared to bulk DNA-Seq. (**B**) In silico bulk DNA-Seq of mitotic abnormalities in embryos with mosaic TE (*n* = 41). Each dot represents the percentage of cells that were affected per mitotic abnormality in the TE of each embryo. The horizontal line at 20% marks the detection limit of bulk DNA-Seq. Complex abnormal cells were excluded.

To investigate whether single-cell sequencing improves detection of chromosomal mosaicism over bulk sequencing approaches, we performed a reanalysis of our single-cell data to mimic the results if the TE of the embryos had been analyzed by bulk DNA sequencing (DNA-Seq) (Figure 3A). To this end, we determined the percentage of TE cells that contained a given mitotic abnormality for each mosaic embryo (*n* = 41). We took into account the copy number states and products of reciprocal events, which compensate for each other when analyzing bulk DNA-Seq data. We then assessed how often at least 20% of TE cells per embryo showed a specific abnormality, to mimic the most sensitive threshold of what bulk DNA-Seq approaches for PGT-A are expected to detect. Our analysis showed that only 20% (*n* = 29 of 147) of all mitotic abnormalities observed in TE cells would have been identified by bulk DNA-Seq (Figure 3B). We therefore conclude that current PGT-A practices lead to a substantial underestimation of mosaicism in the embryo.

### Incidence of structural and chromosome-specific abnormalities.

**Figure 4:**
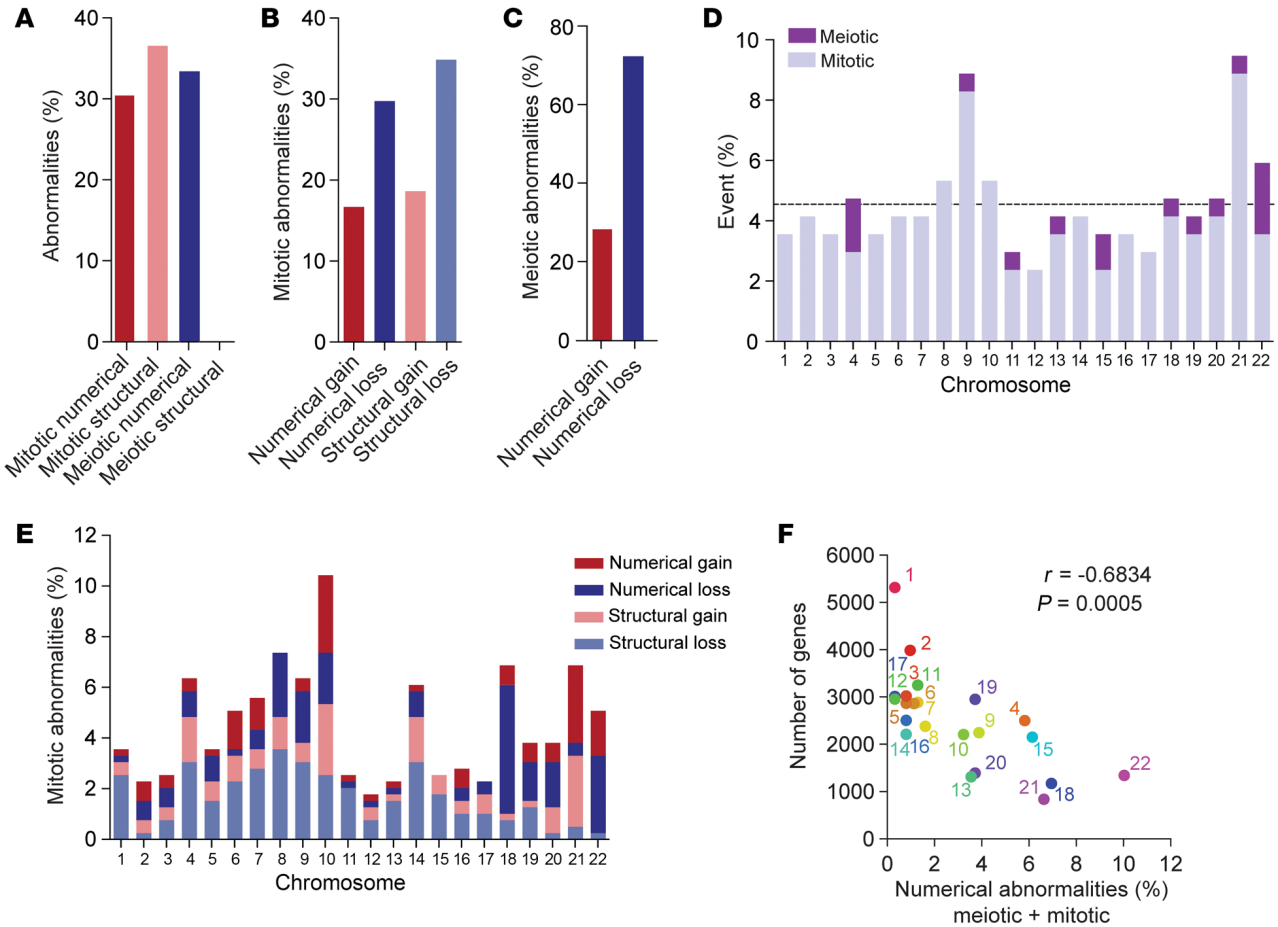
Type of chromosomal abnormalities. (**A**) Percentage of numerical and structural abnormalities of mitotic and meiotic origin (*n* = 618 abnormalities). (**B**) Percentage of mitotic abnormalities involving a whole or partial chromosomal loss or gain (*n* = 413 abnormalities). (**C**) Percentage of meiotic abnormalities with a whole chromosome loss or gain (*n* = 205 abnormalities). (**D**) Percentage of mitotic and meiotic events per chromosome (*n* = 169 events). Horizontal line depicts the expected chance to find an abnormality based on random involvement in an event (1 of 22 = 4.54%). (**E**) Percentage of mitotic abnormalities per affected chromosome (*n* = 413). Colors represent the type of abnormality (numerical or structural gain/loss). (**F**) Graph comparing the mean percentage of total numerical abnormalities and the number of genes per chromosome (Pearson’s correlation coefficient). The X/Y chromosomes were not included in the analysis for chromosome-specific differences. Complex cells and cells that could not be categorized in the above-mentioned groups were excluded.

To determine the frequency of numerical and structural abnormalities, we compared the percentage of abnormalities based on type (numerical/structural) and origin (meiotic/mitotic) (Figure 4A). Structural abnormalities were detected in 69% (*n* = 38 of 55) of the embryos, and the percentage of cells with structural abnormalities was comparable to that of cells with mitotic numerical abnormalities. Interestingly, structural abnormalities were all likely of mitotic origin, as they were always encountered in a low proportion of cells. The length of the chromosomal segments involved ranged from 6.9 to 164 Mb (Supplemental Table 3). Numerical and structural losses were more frequently observed than gains (Figure 4, B and C).

We also investigated the propensity of specific chromosomes to participate in mitotic and/or meiotic errors. The frequency for each chromosome was comparable, except for chromosomes 9, 12, and 21, which were more (chr9, chr21) or less (chr12) frequently involved in an error event (Figure 4D). Moreover, there seemed to be chromosome-specific differences per type of mitotic abnormality (Figure 4E), with larger chromosomes showing more structural abnormalities and smaller chromosomes showing more numerical abnormalities. The incidence of mitotic and meiotic numerical abnormalities per chromosome was significantly inversely correlated with the number of genes on that chromosome (Figure 4F).

### Insights into underlying mechanisms of mitotic error events.

**Figure 5:**
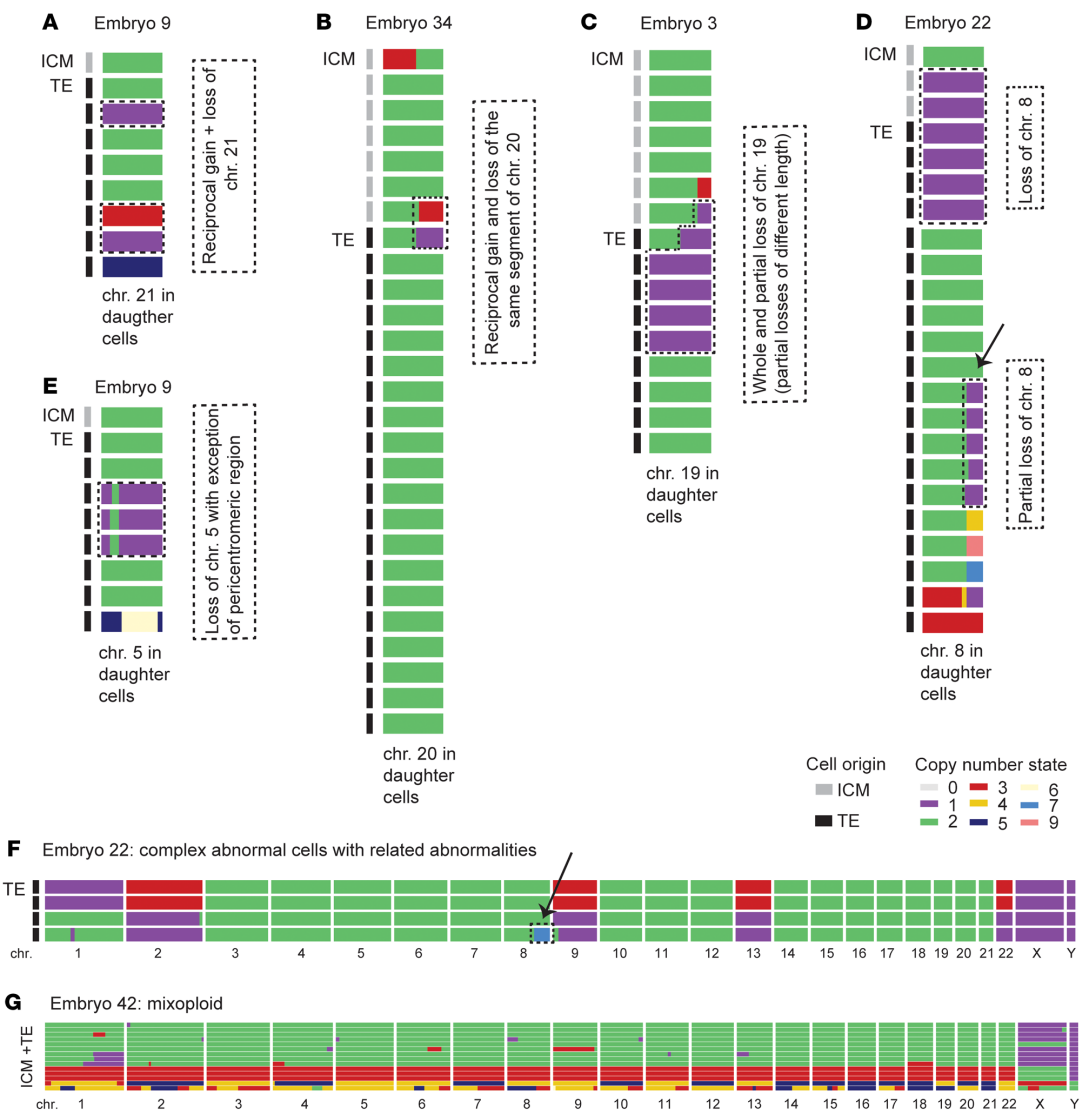
Examples of mitotic error events leading to chromosomal mosaicism observed in different embryos. (**A**–**E**) Copy number of the affected chromosome in all daughter cells within each embryo. (**F** and **G**) Every row represents the scKaryo-Seq result of a single cell and every column represents a different chromosome. The black and gray bars on the left indicate the cell origin (TE or ICM). The colors portray copy number states. The embryo numbers refer to Supplemental Table 3. (**A**) Embryo 9: Reciprocal gain and loss of chr21 in different daughter cells. (**B**) Embryo 34: The reciprocal gain and loss of the same segment of chr20 distributed over the daughter cells implies the occurrence of chromosome breakages. (**C**) Embryo 3: Whole chromosome and partial loss of chr19. The partial losses are of a different length and belong to cells that passed the quality control. (**D**) Embryo 22: There are cells with a whole chromosome or partial loss of chr8 possibly originating from 1 mitotic error. (**E**) Embryo 9: Chr5 is lost with the exception of the pericentromeric region, suggesting (peri) centromeric breakage events. (**F**) Complex abnormal cells within embryo 22 with related chromosomal abnormalities, as in all cells the same chromosomes are affected. One complex abnormal cell also contains multiple copies of a partial gain of chr8, which is most likely the reciprocal product of the partial losses of chr8 observed in other daughter cells (see arrow). (**G**) Embryo 42: Mixoploid chromosomal constitution in which fully triploid cells are present next to diploid cells. The TE and ICM were not analyzed separately in this embryo.

Although aneuploidy is common in human embryos, the underlying mechanisms remain poorly understood. Detailed insight into the karyotype of every cell within each embryo provides important clues as to mitotic error events that lead to chromosomal instability. We identified embryos with similar patterns in chromosomal abnormalities (Supplemental Table 4). We detected 8 events of reciprocal loss and gain of whole chromosomes within daughter cells (Figure 5A). These could originate from 1 missegregation event due to improper functioning of chromosome segregation mechanisms as previously implicated in embryo aneuploidy (6, 36, 37). We observed reciprocal loss and gain of chromosomal segments in 19 events (Figure 5B). This could be the result of incomplete DNA replication and dsDNA breaks before mitosis, which was recently identified as an important contributing factor to chromosome breakage and segmental chromosome errors in human embryos (38, 39) (Figure 5, B–E). We observed whole and partial loss of the same chromosome within daughter cells in 8 events, which could be attributed to breakages of missegregating chromosomes during cytokinesis (40), resulting in segmental losses of different length and finally leading to complete chromosome loss in subsequent cell cycles (41, 42) (Figure 5, C and D). In a particular case, only the pericentromeric region of the chromosome was retained (Figure 5E). Furthermore, lost chromosomes/chromosome segments were shown to frequently end up in extracellular micronuclei that can be reabsorbed by neighboring cells (43–45), explaining some complex chromosomal profiles (Figure 5, D–F). Fully abnormal embryos with different genome-wide abnormalities within daughter cells can result from a noncanonical first cleavage division, whereby parental genomes can segregate into distinct blastomeres and result in mixoploid embryos (46, 47) (Figure 5G).

## Discussion

In this study, we used good-quality human blastocysts, in which only those of good morphological quality were biopsied and analyzed. Despite this, we observed that almost all blastocysts were mosaic. Although our data indicate that 11% of the embryos were cytogenetically normal, it is likely that, in reality, this proportion is even lower, given the relatively small number of cells sequenced from these embryos and the possibility that some of the nonsequenced cells were abnormal. Our results lend strong support to the notion that mosaicism is a common feature of early human development (1, 27).

Our knowledge of the high rates of aneuploidy in preimplantation embryos derives mainly from embryos generated by in vitro fertilization (IVF), raising the question of whether this aneuploidy is related to patient characteristics or specific parts of the IVF procedure. Analysis of large PGT-A data sets has so far not resulted in consistent identification of contributing factors (reviewed in ref. 15). Importantly, a comparison of in vitro– and in vivo–generated blastocysts found no differences in aneuploidy or mosaicism rates (48). Mosaicism after natural conception may also be underestimated, as recently higher rates were observed in first trimester miscarriages when multiple site sampling was performed (49). Overall, evidence from miscarriages, chorionic villus sampling, and analysis of term placentas shows that mosaicism associated with IVF persists beyond the preimplantation embryo at a rate similar to that associated with spontaneous conception (50–52).

Studies have demonstrated that PGT-A–tested embryos with low proportions of abnormal cells have clinical outcomes similar to those of chromosomally normal embryos (17, 53, 54). In our data set, most embryos were diploid-aneuploid mosaic, in which the proportion of normal cells was higher than the proportion of abnormal cells. These embryos may have the potential to develop normally (55). However, it is important to note that not every embryo that tested chromosomally normal after PGT-A results in a healthy live birth. Additionally, there are reports in which embryos with a uniform abnormal PGT-A result led to a successful pregnancy (54, 56). Undetected mosaicism or a TE biopsy that was not representative of the remaining embryo due to mosaicism could provide explanations for individuals with unexpected clinical outcomes after the transfer of PGT-A–tested embryos.

Our cytogenetic findings are a snapshot of the chromosomal composition of blastocysts at the moment of biopsy, which is predetermined by the timing of the mitotic error event during embryo development, chromosome-specific differences in segregation error bias, and/or selective pressure against (specific) chromosomal abnormalities. Human embryos are especially prone to mitotic errors during cleavage divisions (1, 4, 6–8, 10), however, the underlying mechanisms are poorly understood. Factors such as maternal mRNA transcripts and proteins, shortened cell cycles, altered spindle dynamics, and permissive cell-cycle control mechanisms, such as the spindle assembly checkpoint (SAC), might be involved (reviewed in ref. 37). Although aneuploidy is observed to decrease toward the blastocyst stage (11, 12), in our cohort we observed daughter cells of the same mitotic error event to be restricted to either the TE or ICM in a high proportion of embryos. This suggests that these mitotic error events may have occurred after embryonic lineage specification. Cell-cycle control mechanisms might still be relaxed at the blastocyst stage, permitting mitotic errors. This is in line with a study using gene knockdown and pharmacological approaches to assess SAC strength in in vitro–cultured mouse embryos (57). In morula-stage embryos, misaligned chromosomes were able to mount a SAC signal, but this was unable to prevent the onset of anaphase. This suggests that molecular mechanisms controlling chromosome segregation may not only be permissive during the first cell divisions before embryonic genome activation, but also at the morula stage. In addition, it was recently shown that mouse and human blastocyst expansion causes TE cell nuclear budding and DNA shedding, giving rise to micronuclei (58). The frequency of aneuploidy and mosaicism in cattle is similar to that in humans (59), and the spontaneous presence of micronuclei in TE cells has also been reported in bovine blastocysts (45), indicating that this mechanism may be conserved.

We further showed that all chromosomes can be involved in a mitotic error, with loss of chromosomal content being more prevalent than gain for both mitotic and meiotic abnormalities. This contradicts previous research in large PGT-A data sets of multicellular biopsies, which reported similar frequencies of chromosomal gains and losses (12, 60). Recently, experimentally induced replication fork stalling in human embryos was shown to lead to both structural and numerical abnormalities, with substantially more DNA loss than gain (38, 61). In the end, only chromosomal gain is compatible with development to term, with the exception of chromosome X (3, 62). Thus, selective pressures that possibly eliminate embryos or cells with chromosomal loss after implantation (63) might still not be fully active at the blastocyst stage. Evidence in human embryos and gastruloids at peri- and post- implantation stages of development suggest that this selection is the result of autophagy-mediated apoptosis that eliminates aneuploid cells, while diploid cells show increased proliferation (27, 60, 63, 64). In addition, some evidence exists that the embryo-endometrium dialogue also contributes to the elimination of embryos with chromosomal loss (reviewed in ref. 65).

We found the proportion of abnormal cells in TEs and ICMs to be comparable without showing a preferential allocation toward either one of the lineages. This is in line with single-cell observations in blastocysts (27) and bulk DNA analysis of multicellular samples of ICMs versus TEs (16, 31). However, we did observe complex abnormal cells more frequently in the TE, suggesting some bias in selective pressure for such cells between the 2 lineages. This selective pressure may increase in time, as the proportion of abnormal cells in the ICM was reported to be reduced somewhat later in development, between post-fertilization days 7 and 14 (27).

Using scKaryo-Seq, we accurately identified structural abnormalities on a single-cell level, revealing a 2-fold higher incidence than previously reported (10, 19–21). In our cohort, structural abnormalities were of mitotic origin and mostly concerned the loss of a chromosomal segment. This again points to an important role for replication stress and/or DNA damage driving chromosomal instability in preimplantation embryos (38, 40). Interestingly, in several embryos, we observed mosaicism for terminal losses of different length was seen. Similar observations have been made in first trimester chorionic villi and term placentas of pregnancies from in vivo conception, in which the fetus itself had one of the deletions (41, 42, 66). The high incidence of mosaic structural chromosome abnormalities in our cohort indicates that the structural abnormalities observed during pregnancy may have originated during the preimplantation period and that this also occurs in vivo.

Our study has some limitations. Human embryos donated for research are scarce, and therefore our findings are based on a limited set of 55 embryos. Our analysis focused on good-quality blastocysts, and it is possible that poorer-quality blastocysts might exhibit different patterns of abnormalities. Because of ethical restrictions, the embryos used in this study were all frozen/thawed, and we cannot exclude a potential effect of the freezing process on the incidence of mosaicism. However, previous findings using single-cell RNA-Seq data for comprehensive karyotyping also included results from fresh blastocysts. In line with our observations, all embryos had at least 1 chromosomally abnormal cell, and 84% of the day-5 to day-7 blastocysts were found to be mosaic (27, 67). A technical limitation is that only a proportion of the cells in each embryo could be successfully karyotyped, as isolation and cytogenetic analysis of viable single cells at the blastocyst stage is technically challenging. Improvement herein is crucial for future research. Still, the proportion of normal cells within mosaic blastocysts correlated with embryo developmental characteristics, indicating that the sample of successfully karyotyped cells was representative for the constitution of the whole embryo. The asynchrony in DNA replication domains during the S-phase of the cell cycle could cause overcalling of the structural abnormalities in single-cell analysis (68). However, in most cases we observed several cells with the same structural abnormality within 1 embryo, making S-phase artifacts unlikely. Moreover, using SNP-based haplotyping would have been a more accurate way to distinguish meiotic from mitotic errors (69, 70). This would also have allowed the identification of uniparental disomy (71), potentially revealing an even higher incidence of chromosomal abnormalities.

Our single-cell analysis approach provides a comprehensive view of the chromosomal constitution of good-quality human blastocysts and contributes to an improved understanding of mechanisms leading to mosaicism. In current PGT-A practice, chromosomal mosaicism is diagnosed after bulk analysis of a TE biopsy by the observation of an intermediate chromosome copy number on a NGS profile. Our results show that this approach is likely to underestimate mosaicism and generate false-negative, but potentially also false-positive, results. The interpretation of PGT-A results therefore warrants caution, and patients proceeding to PGT-A should be counseled about the technical and biological limitations. To better understand the clinical consequences of mosaicism at the blastocyst stage, future research should aim at elucidating the effect of mosaicism on embryo development and on the fate of chromosomally abnormal cells during further development.

## Methods

### Embryo warming and culturing.

Ovarian stimulation, oocyte retrieval, IVF procedures, assessment of embryo morphology, and cryopreservation were performed between 2013 and 2015, as described previously (72, 73). During this period, embryos were routinely cryopreserved, as described previously, at the morula stage and selected for cryopreservation when embryos had at least 12 cells or showed at least 30% compaction (74). Embryos were anonymously donated by couples who provided consent for use in research. The median maternal age of the embryo donors was 34 years (range, 22–42 years). All samples were deidentified prior to the thawing process, and therefore clinical and treatment information was not available.

Donated embryos were thawed by placing the embryo straw (CBS High Security, Cryo Bio System) at room temperature. After release from the straw, the embryo was warmed using the RapidWarm Omni kit (Vitrolife), according to the manufacturer’s instructions. After thawing, each embryo was placed in a well of an EmbryoSlide (Vitrolife) culture dish containing 25 μL G-TL culture medium (Vitrolife) under 1.4 mL mineral oil (Gynemed) and cultured in an EmbryoScope time-lapse incubator (Vitrolife) until the blastocyst stage (16) (Figure 1A). Assessment of blastocyst morphology was performed in accordance with the standardized scoring system established by the European Society of Human Reproduction and Embryology (ESHRE) consensus (72). Blastocyst expansion was assessed on a scale of 1 (no expansion) to 6 (fully hatched embryo). ICMs and TEs were scored on a scale of 1 to 3, with 1 representing the highest quality (Supplemental Tables 2 and 3). Only blastocysts with an expansion grade of at least 3 were considered for biopsy (as shown in Supplemental Videos 1–3). A biopsy was not performed in cases of low blastocyst quality (grade 3 for ICMs and/or TEs) or when the ICM was crescent shaped and flattened to the TE.

### Time-lapse imaging and blastocyst surface measurements.

The EmbryoScope time-lapse incubator automatically captures images every 10 minutes, whereas the EmbryoViewer software (Vitrolife) provides an ellipse tool with which the surface of the blastocyst can be measured in square micrometers. We performed measurements every hour, starting from the first image of blastocoel formation. The ellipse was formed around the outer edge of the trophectoderm, excluding the zona pellucida. To calculate the expansion rate of each blastocyst, we identified the moment at which the blastocyst achieved its maximum surface area, then subtracted the initial surface area, and divided this value by the number of measurements taken during the time interval between these points (34).

### Disaggregation of ICM and TE cells of human blastocysts.

Embryo disaggregation was performed within 9–32 hours from the start of blastulation. First, the ICM was isolated from the TE (Figure 1A) as previously described (16). For disaggregation of the cells, the ICM and TE were placed in separate droplets of Accutase Cell Detachment Solution (MilliporeSigma) for 10 minutes at 37°C. The remaining clusters of cells were disaggregated mechanically with the assistance of the biopsy pipette. The single cells were placed manually with a 170 μL EZ-Squeeze pipette (Cooper Surgical) in a volume of approximately 1 μL Accutase into a 384-well plate under oil, which was kept on ice between transfers. Once filled, the plate was centrifuged at 2,000*g* for 1 minute and stored at –20°C until scKaryo-Seq.

### scKaryo-Seq and quality control.

ScKaryo-Seq was performed as described previously with a few modifications (32). To each well, 200 nL lysis buffer (6× Cutsmart, New England BioLabs) and 1.8 μg Proteinase K (Thermo Fisher Scientific) were added. This was incubated for 2 hours at 55°C and heat inactivated for 10 minutes at 80°C. Genomic DNA was digested using 100 nL digestion mix (1× CutSmart and 0.1 U NlaIII, New England BioLabs) at 37°C for 2 hours and 80°C for 20 minutes. A volume of 100 nL of 500 nM NlaIII-specific adapters was dispensed in each well followed by 400 μL of an adapter ligation mix consisting of 1× ligase buffer, 3.33 mM ATP, and 4,000 U of T4 DNA ligase (all from New England BioLabs). The rest of the protocol was performed as described previously (32), where, in short, DNA fragments were amplified using in vitro transcription. Next, the RNA was reverse transcribed back into DNA, and Illumina-compatible adapters were added with PCR. Libraries were sequenced × 75 bp using an Illumina Next-Seq 500. Copy number analysis and quality control were performed with AneuFinder software (75). ScKaryo-Seq of lymphocytes from healthy donors and BJ-hTERT cells were used as a diploid reference to determine variable bin sizes. Copy numbers were called using the divisive algorithm. Cells with a minimum total read count of 15,000, a maximum spikiness score of 0.25, and a minimum Bhattacharyya score of 0.65 passed quality control. Although scKaryo-Seq accurately detects copy number variations in flow-sorted cells (32, 33), we first confirmed that this was also the case for single fetal cells deposited manually in a 384-well plate. For this, we used 7 different fetal cell lines with known numerical and structural abnormalities as positive controls (Supplemental Methods). Fetal cell lines were cultured in Chang D medium (Irvine Scientific) and 1% penicillin-streptomycin (Gibco, Thermo Fisher Scientific). To quantify segregation errors and micronuclei of these cells, the cells were plated on 12 mm round glass coverslips (Superior Marienfeld, Thermo Fisher Scientific) and fixed using 4% paraformaldehyde (MilliporeSigma) 1–2 days after splitting. Cells were washed 4 times with PBS and permeabilized for 10 minutes with 0.1% Triton X-100 (MilliporeSigma) in PBS. Next, cells were stained with DAPI for 1 minute, washed twice with PBS, and mounted using ProLong Gold Antifade (Thermo Fisher Scientific) on a glass slide. Imaging was performed on a DeltaVision RT system (Applied Precision/GE Healthcare) with a ×1.40/60 numerical aperture (NA) UplanSApo objective (Olympus) as *Z*-stacks at 0.5 μm intervals. For deconvolution, SoftWorx (Applied Precision/GE Healthcare, version 6.5.2) was used. Image analysis and quantification was performed using Fiji ImageJ, version 2.0.0 (NIH). 

### Interpretation of scKaryo-Seq results and definitions.

We routinely checked 4 control parameters: the quality of the sequencing plot, the total read count, the spikiness score, and the Bhattacharyya score. If a cell showed a borderline result for 1 of the quality control parameters, the quality of the sequencing plot as assessed by visual inspection by 3 independent observers was decisive. If the inspection clearly showed the same abnormality as another cell within the same embryo, it was considered a true finding. Furthermore, we distinguished meiotic from mitotic errors on the basis of the proportion of abnormal cells with a specific abnormality. An abnormality observed in every cell within an embryo was considered a result of a meiotic error, whereas an abnormality observed in part of the cells was considered to originate from a mitotic error. Cells with more than 4 abnormalities were categorized as complex abnormal. Abnormal cells in which the same chromosome(s)/chromosomal segments were affected were considered products of 1 mitotic error event, and this was used to calculate the number of mitotic events per mosaic embryo.

### Statistics.

Statistical analysis and preparation of graphs were conducted using GraphPad Prism, version 8.4.3 (GraphPad Software). Data are presented as the mean ± SEM. The applied tests are indicated in the figure legends. Significance was determined with a 2-sided Fisher’s exact test. Correlation of variables was tested with linear regression or Pearson’s correlation coefficient. *P* values of less than 0.05 were considered statistically significant.

### Study approval.

Surplus cryopreserved good-quality human preimplantation embryos of unknown chromosomal constitution were donated with written informed consent from patients who underwent IVF treatment at the Erasmus University Medical Center Rotterdam. Almost all embryo donors fulfilled their wish for a child and no longer wished to use their frozen surplus embryos to establish another pregnancy. The use of these embryos for this study, including low-pass genome sequencing and controlled access database submission, was approved by the Dutch Central Committee on Research Involving Human Subjects (CCMO, The Hague, Netherlands, NL82597.000.22) and the METC ethics committee (Erasmus MC, Rotterdam, Netherlands).

### Data availability.

The raw sequencing data were deposited in a controlled-access repository, the European Genome-Phenome Archive (EGA) (accession number PRJEB68313). Values for all data points in graphs are reported in the Supplemental Supporting Data Values file.

## Author contributions

EBB designed the study. EAC performed the biopsies and prepared the plates for scKaryo-Seq. SJK performed the sequencing. EAC and SJK analyzed the data. All authors interpreted the data. EAC and SJK drafted the manuscript. EBB, DVO, JSEL, and GJPLK performed critical revision of the manuscript. All authors gave approval for publication of the present version of this manuscript. The order of the authors was assigned on the basis of their efforts and contributions to the study.

## Supplementary Material

Supplemental data

Supplemental video 1

Supplemental video 2

Supplemental video 3

Supporting data values

## Version 1 01/04/2024

In-Press Preview

## Version 2 03/15/2024

Electronic publication
