# Corrigendum to Single-cell DNA sequencing reveals a high incidence of chromosomal abnormalities in human blastocysts

**DOI:** 10.1172/JCI210864

**Published:** 2026-08-17

**Authors:** Effrosyni A. Chavli, Sjoerd J. Klaasen, Diane Van Opstal, Joop S.E. Laven, Geert J.P.L. Kops, Esther B. Baart

Original citation: *J Clin Invest*. 2024;134(6):e174483. https://doi.org/10.1172/JCI174483

Citation for this corrigendum: *J Clin Invest*. 2026;136(16):e210864. https://doi.org/10.1172/JCI210864

The *Data* *availability* section has been updated to provide the correct accession number for the raw sequencing data. The corrected text is below.

*Data availability*. The raw sequencing data were deposited in a controlled-access repository, the European Genome-Phenome Archive (EGA) (accession number EGAS50000001913). Values for all data points in graphs are reported in the Supplemental Supporting Data Values file.

The authors regret the error.

The HTML and PDF versions have been updated online.

## Version 1 08/17/2026

Electronic publication

