# Supplementary material for: Single-cell DNA sequencing reveals a high incidence of chromosomal abnormalities in human blastocysts
Source: J Clin Invest. 2024 Jan 4;134(6):e174483. doi: 10.1172/JCI174483 (PMC10940095; doi:10.1172/JCI174483)
Supplement: Supplemental data [file jci-134-174483-s204.pdf]

Supplemental Methods

Validation of scKaryo-seq for detection of structural and numerical chromosomal abnormalities in single fetal cells deposited manually in a 384 well plate

This file includes:

- Material and Methods
- Supplemental Table 1

We first performed scKaryo-seq on manually sorted cells from frozen-thawed cell lines obtained from amniotic fluid or chorionic villi cell cultures with known numerical chromosomal abnormalities (45,X, 47,XX,+21, 47,XX,+18, 47,XY,+13) and with structural abnormalities involving chromosomal segments of different length: 46,XX,der(15)t(1;15)(q32.1;p11) resulting in a 48Mb gain of 1q32.1->qter; 46,XY,der(9)t(4;9)(q33;p24) resulting in a 24.5Mb gain of 4q33->qter and a 4.2 Mb loss of 9p24->pter and 46,XY,der(13;20)(p10;q10),+20, resulting in a 27 Mb gain of 20p. The accuracy of scKaryo-seq to identify the expected copy number state of each chromosome was calculated as previously described (1-3) and was overall found to be 99.5% (Supplemental Table 1). In only 3 out of 376 cells (<1%) the expected abnormality was not detected and 28 out of 376 cells presented additional abnormalities next to the expected one (7.4%). The additional chromosomal abnormalities consisted of abnormalities that were observed in single cells only within each cell line and are likely of biological origin for the following reasons: the technical error rate for scKaryo-seq is expected to be extremely low, as copy number alterations are called for regions spanning multiple bins (~5Mb) of reads. The likelihood that series of neighboring bins all have the same error is extremely unlikely. Also, we and others have reported occasional mis-segregations of chromosomes in normal, healthy cell lines (4-6), which very likely lead to aneuploidy. To verify if this also counts for chorionic villi cell lines, we fixed and DAPI-stained cells from two chromosomally normal and one abnormal chorionic villi cell line and we observed different levels of mis-segregations and micronuclei in each cell line (Supplemental Figure 1). This confirms experiences from prenatal cytogenetic diagnosis, where it is known that culturing of chorionic villi and amniotic fluid cells used for karyotyping metaphases, may introduce chromosome aberrations in a subset of cells, known as culture artefacts (pseudomosaicism). In addition,

abnormalities have been observed in normal healthy human cells ranging from 3%-40% depending on the tissue (7,8). Hence, we conclude that our embryo-customized scKaryo-seq approach allows us to accurately detect both numerical and unbalanced structural chromosomal abnormalities.

Karyotype	Cells			Chromosomes				
	Nº of karyotyped cells	Nº cells with the expected abnormality + additional abnormalities	Nº of cells without the expected abnormality	Nº of expected abnormalities	Nº of diploid chromosomes expected	Nº of known abnormalities detected	Nº of diploid chromosomes detected	Accuracy
45,X	81	10	0	81	1782	81	1767	99,2%
47,XX,+21	94	6	1	94	2068	93	2062	99,7%
47,XX,+18	62	2	0	62	1364	62	1360	99,7%
47,XY,+13	34	3	0	34	748	34	741	99,1%
46,XX,der(15)t(1;15)(q32.1;p11)	21	0	0	21	462	21	462	100,0%
46,XY,der(9)t(4;9)(q33;p24)	23	3	0	23	506	23	502	99,2%
46,XY,der(13;20)(p10;q10),+20	61	4	2	61	1342	59	1337	99,5%
total	376	28	3	376	8272	373	8231	99,5%

Supplemental Table 1: Results from scKaryo-seq validation experiments for identification of the expected copy number state of each chromosome, using cultured amniotic fluid and chorionic villi cells with a known abnormal karyotype. Accuracy= chromosomes with the expected copy number state / total number of chromosomes. Nº: number

1. Kung, A., Munné, S., Bankowski, B., Coates, A., and Wells, D. (2015). Validation of next-generation sequencing for comprehensive chromosome screening of embryos. Reprod. Biomed. Online *31*, 760–769.

2. Wells, D., Kaur, K., Grifo, J., Glassner, M., Taylor, J.C., Fragouli, E., and Munne, S. (2014). Clinical utilisation of a rapid low-pass whole genome sequencing technique for the diagnosis of aneuploidy in human embryos prior to implantation. J. Med. Genet. *51*, 553–562.

3. Capalbo, A., Poli, M., Rienzi, L., Girardi, L., Patassini, C., Fabiani, M., Cimadomo, D., Benini, F., Farcomeni, A., Cuzzi, J., et al. (2021). Mosaic human preimplantation embryos and their developmental potential in a prospective, non-selection clinical trial. Am. J. Hum. Genet. *108*, 2238–2247.

4. Janssen, A., van der Burg, M., Szuhai, K., Kops, G.J.P.L., and Medema, R.H. (2011). Chromosome segregation errors as a cause of DNA damage and structural chromosome aberrations. Science *333*, 1895–1898.

5. Bolhaqueiro, A.C.F., Ponsioen, B., Bakker, B., Klaasen, S.J., Kucukkose, E., van Jaarsveld, R.H., Vivié, J., Verlaan-Klink, I., Hami, N., Spierings, D.C.J., et al. (2019). Ongoing chromosomal instability and karyotype evolution in human colorectal cancer organoids. Nat. Genet. *51*, 824–834.

6. Thompson, S.L., and Compton, D.A. (2008). Examining the link between chromosomal instability and aneuploidy in human cells. J. Cell Biol. *180*, 665–672.

7. Knouse, K.A., Wu, J., Whittaker, C.A., and Amon, A. (2014). Single cell sequencing reveals low levels of aneuploidy across mammalian tissues. Proceedings of the National Academy of Sciences *111*, 13409–13414.

8. McConnell, M.J., Lindberg, M.R., Brennand, K.J., Piper, J.C., Voet, T., Cowing-Zitron, C., Shumilina, S., Lasken, R.S., Vermeesch, J.R., Hall, I.M., et al. (2013). Mosaic copy number variation in human neurons. Science *342*, 632–637.

Embryo nr.	Morphology*	ICM cells	Karyotyped ICM cells	%Karyotyped ICM cells	TE cells	Karyotyped TE cells	%Karyotyped TE cells	Total cells	Total karyotyped cells	% karyotyped cells
**Embryos with successful karyotyping of cells from TE and ICM**										
1	B411	6	3	50%	35	17	49%	41	20	49%
2	B422	2	1	50%	45	24	53%	47	25	53%
3	B412	10	7	70%	16	9	56%	26	16	62%
4	B512	12	7	58%	33	20	61%	45	27	60%
5	B412	7	4	57%	31	5	16%	38	9	24%
6	B411	14	5	36%	72	56	78%	86	61	71%
7	B411	11	9	82%	67	58	87%	78	67	86%
8	B412	6	4	67%	28	11	39%	34	15	44%
9	B421	3	1	33%	19	5	26%	22	6	27%
15	B411	9	2	22%	35	8	23%	44	10	23%
16	B411	9	8	89%	36	19	53%	45	27	60%
17	B411	19	4	21%	44	18	41%	63	22	35%
18	B412	12	6	50%	28	16	57%	40	22	55%
19	B411	16	10	63%	52	50	96%	68	60	88%
20	B412	6	5	83%	29	24	83%	35	29	83%
21	B412	4	3	75%	50	21	42%	54	24	44%
22	B411	8	3	38%	41	20	49%	49	23	47%
23	B322	1	1	100%	17	3	18%	18	4	22%
24	B412	6	5	83%	37	34	92%	43	39	91%
25	B411	11	1	9%	57	29	51%	68	30	44%
27	B412	19	7	37%	49	9	18%	68	16	24%
28	B411	9	1	11%	43	21	49%	52	22	42%
29	B412	7	3	43%	47	17	36%	54	20	37%
30	B421	6	1	17%	43	5	12%	49	6	12%

31	B411	11	2	18%	61	18	30%	72	20	28%
32	B322	8	1	13%	33	11	33%	41	12	29%
33	B412	6	1	17%	23	5	22%	29	6	21%
34	B411	17	8	47%	38	19	50%	55	27	49%
35	B412	4	4	100%	35	20	57%	39	24	62%
36	B412	8	4	50%	34	10	29%	42	14	33%
40	B411	5	2	40%	41	9	22%	46	11	24%
41	B412	3	2	67%	28	23	82%	31	25	81%
45	B422	7	1	14%	39	13	33%	46	14	30%
46	B411	9	3	33%	43	19	44%	52	22	42%
47	B312	7	4	57%	8	2	25%	15	6	40%
48	B412	4	2	50%	30	8	27%	34	10	29%
51	B422	3	1	33%	11	2	18%	14	3	21%
52	B522	1	1	100%	16	3	19%	17	4	24%
53	B312	12	2	17%	26	6	23%	38	8	21%
54	B422	3	1	33%	15	3	20%	18	4	22%
55	B412	14	7	50%	23	3	13%	37	10	27%
**Embryos with successful karyotyping of only TE**										
10	B312	N/A	N/A	N/A	31	13	42%	31	13	42%
11	B411	N/A	N/A	N/A	59	40	68%	59	40	68%
14	B511	N/A	N/A	N/A	53	16	30%	53	16	30%
26	B422	N/A	N/A	N/A	36	13	36%	36	13	36%
38	B422	N/A	N/A	N/A	20	18	90%	20	18	90%
39	B421	N/A	N/A	N/A	18	13	72%	18	13	72%
43	B421	N/A	N/A	N/A	45	12	27%	45	12	27%
44	B522	N/A	N/A	N/A	43	6	14%	43	6	14%
49	B422	N/A	N/A	N/A	28	8	29%	28	8	29%

**Embryos with successful karyotyping of only ICM**										
37	B521	7	5	71%	N/A	N/A	N/A	7	5	71%
50	B412	8	8	100%	N/A	N/A	N/A	8	8	100%
**ICM and TE not separated**										
12	B422	N/A	N/A	N/A	N/A	N/A	N/A	65	33	51%
13	B422	N/A	N/A	N/A	N/A	N/A	N/A	67	39	58%
42	B521	N/A	N/A	N/A	N/A	N/A	N/A	49	13	27%
**MEDIAN**				**50%**			**38%**			**42%**

Supplemental Table 2: Total number of TE and ICM cells that were retrieved and total number of cells with a successful cytogenetic analysis per embryo. TE (Trophectoderm), ICM (Inner cell mass), N/A (not available). * Morphological grading according to the Alpha Scientists in Reproductive Medicine and ESHRE Special Interest Group of Embryology consensus on embryo assessment (1).

1. Scientists A, ESHRE Special Interest Group of Embryology. The Istanbul consensus workshop on embryo assessment: proceedings of an expert meeting†. Hum Reprod. 2011;26(6):1270–1283.

**Embryo no.**	**Morphology***	**Lineage**	**Karyotype**	**Structural abnormality genomic location start-end (Mb)**	**Nºof cells**
	**Diploid-aneuploid mosaic**				
**1**	B411	TE	2N,XX		12
			2N,XX,-22		3
			complex		2
		ICM	2N,XX		2
			2N,XX,-22		1
**2**	B422	TE	2N,XY		18
			2N,XY,-14		3
			2N,XY,-22		2
			complex		1
		ICM	2N,XY		1
**3**	B412	TE	2N,XX		3
			2N,XX,-19		5
			2N,XX,-S19	28-57	1
		ICM	2N,XX		5
			2N,XX,+S19	45-57	1
			2N,XX,-S19	45-57	1
**4**	B512	TE	2N,XY		10
			2N,XY,-18		10
		ICM	2N,XY,-18		6
			2N,XY,+4,-18		1
**5**	B412	TE	2N,XY		1
			2N,XY,+22		4
		ICM	2N,XY		2
			2N,XY,+22		2
**6**	B411	TE	2N,XY		40
			2N,XY,-5		1
			2N,XY,-S15	43-101	1
			complex		14
		ICM	2N,XY		5
**7**	B411	TE	2N,XY		52
			2N,XY,-S9	1-38(~)	3
			2N,XY,-S10	75-132	1
			complex		2
		ICM	2N,XY		7
			2N,XY,-S9	1-38	2
**8**	B412	TE	2N,XX		9
			2N,XX,-S8	79-144	1

			2N,XX,+S2,-S8,+16,+18	44-119, 62-126	1
		ICM	2N,XX		4
**9**	B421	TE	2N,XY		1
			2N,XY,-S5,-S5	1-25(~), 52-181	3
			2N,XY, -21		2
			2N,XY,+21		1
			complex		1
		ICM	2N,XY		1
**10**	B312	TE	2N,XX		10
			2N,XY,+21		3
		ICM	N/A		
**11**	B411	TE	2N,XX		25
			2N,XX,+7		1
			2N,XX,-7,-9		1
			2N,XX,-3,-7,+19,-22		1
			2N,XX,+7,-S13,-S15	80-111, 62-101	1
			2N,XX,-S13,+22	85-111	1
			2N,XX,-S8	53-144	1
			2N,XX,-S7	129-159 (~)	6
			2N,XX,+S21	1-22	3
			complex		1
		ICM	N/A		
**12**	B422	TE+ICM	2N,XY		15
			2N,XY,-S11	101-133	6
			2N,XY,+21		1
			2N,XY,+6		1
			2N,XY,-8,-17		1
			4N,XXYY,+4,+6		1
			complex		8
**13**	B422	TE+ICM	2N,XY		25
			2N,XY,-9		3
			2N,XY,-S2	123-239	1
			2N,XY,-S16	48-87	3
			2N,XY,-S14	28-105	1
			2N,XY,-S17	64-81(~)	3
			2N,XY,+S4	165-187	1
			complex		3
**14**	B511	TE	2N,XX		7
			2N,XX,-S1	157-247(~)	3
			2N,XX,-S10	1-34	1
			2N,XX,-S11	63-133	2

			2N,XX,+S21	1-22	1
			complex		2
		ICM	N/A		
**15**	B411	TE	2N,XX		6
			2N,XX,-20		1
			2N,XX,-21		1
		ICM	2N,XX		1
			2N,XX,+20		1
**16**	B411	TE	2N,XY		15
			2N,XY,+12		1
			2N,XY,+S3,+6,-S18	1-98, 35-79	1
			complex		2
		ICM	2N,XY		7
			2N,XY,-12		1
**17**	B411	TE	2N,XX		17
			2N,XX,-16		1
		ICM	2N,XX		2
			2N,XX,+16		1
			2N,XX,+S21	1-22	1
**18**	B412	TE	2N,XY		5
			2N,XY,-10,-20		1
			2N,XY,+10,-20		1
			2N,XY,-18,-19,-20		1
			2N,XY,+6		1
			2N,XY,+10		1
			2N,XY,+S2,+S9	1-56, 1-38	1
			4N,XXYY,+6,-10,-20		1
			complex		4
		ICM	2N,XY		3
			2N,XY,-10,-20		1
			complex		2
**19**	B411	TE	2N,XY		39
			2N,XY,-S1	1-85	2
			2N,XY,+S1	1-85	1
			2N,XY,-S10	1-33 (~)	2
			3N,XXY,-S10	33-132	1
			complex		5
		ICM	2N,XY		9
			2N,XY,+5,+10,+21		1
**20**	B412	TE	2N,XX		14
			2N,XX,-S4,-S6	132-187, 87-167	1

			2N,XX,+S4,-S6	142-187, 82-167	1
			2N,XX,-S6	82-167	1
			2N,XX,+S12	7-70	1
			2N,XX,-S14	51-105	1
			2N,XX,-S15	86-100(~)	3
			3N,XXX		1
			3N,XXX,+S14,+S15	51-105, 86-100	1
		ICM	2N,XX		5
**21**	B412	TE	2N,XX		8
			2N,XX,-S6	96-167(~)	2
			2N,XX,+S6	96-167	1
			2N,XX,+S6	1-25	1
			2N,XX,-S6,+S6,+19	1-25, 25-167	1
			3N,XXX,-S3,-S6,+S6	52-197, 1-25, 25-167	1
			2N,XX,-S3,-S10,-S14	1-49, 78-132, 96-105	1
			2N,XX,-S7	1-64(~)	2
			2N,XX,+S7	1-64	1
			2N,XX,+S20	1-32(~)	2
		ICM	2N,XX		2
			2N,XX,+S7	1-28	1
**22**	B411	TE	2N,XY		3
			2N,XY,-8		4
			2N,XY,-S8	105-144(~)	6
			2N,XY,+S8	105-144	2
			2N,XY,-2,-9,-13,-22		1
			complex		4
		ICM	2N,XY,-8		2
			complex		1
**23**	B322	TE	2N,XY		3
			2N,XY,+S9	74-138	1
		ICM	2N,XY,-S9	70-138	1
**24**	B412	TE	2N,XY		15
			2N,XY,-S5,-S19	173-180, 42-58	1
			2N,XY,+S5,-S19	173-180, 42-58	1
			2N,XY,-S5	101-180	2
			2N,XY,-S5, null S5	1-96, 96-180	1
			2N,XY,+S5	1-101	1
			2N,XY,-16		1
			4N,XXYY,+16,-S19	42-58	1
			2N,XY,-S12	102-131	1
			2N,XY,+S12	102-131	1

			2N,XY,+2,+3,+11,+13		1
			complex		8
		ICM	2N,XY		1
			2N,XY,+1,+2,+6		1
			2N,XY,-1,-2,-6		1
			2N,XY,+S5	101-180	1
			complex		1
**25**	B411	TE	2N,XY		24
			2N,XY,-7		1
			2N,XY,-S7	1-54(~)	2
			2N,XY,-S10,+S10,-S15	1-74, 74-132, 1-90	1
			2N,XY,+S10,-S10,+S15,-S15	1-74, 74-132, 1-42, 42-100	1
		ICM	2N,XY,-S10,-S15	71-132, 46-100	1
**26**	B422	TE	2N,XY		3
			2N,XY,-9,-18		2
			2N,XY,-S18	34-79(~)	2
			2N,XY,+S18	1-34	1
			2N,XY,-14		1
			2N,XY,-S14	51-105	1
			2N,XY,-S14,+20	47-105	1
			2N,XY,+S10	1-30	1
			complex		1
		ICM	N/A		
**45**	B422	TE	2N,XY		8
			2N,XY,-S12	106-131	1
			2N,XY,-S1,-S8,-S21	150-246, 89-144, 22-45	1
			2N,XY,+7,+16		1
		ICM	2N,XY		1
**46**	B411	TE	2N,XX		18
			2N,XX,+S13	45-111	1
		ICM	2N,XX		3
**47**	not in embryoscope	TE	2N,XX		2
		ICM	2N,XX		3
			2N,XX,+S7	99-159	1
**48**	B412	TE	2N,XY		5
			2N,XY,-S9,+19	38-138	1
			2N,XY, -2,-S6	33-46	1
			2N,XY,-S5	143-180	1
		ICM	2N,XY		1
			2N,XY,-S6,-S10,-S12	46-68,1-93, 98-109	1

**<u>49</u>**	B422	TE	2N,XX		5
			3N,XXX		1
			2N,XX, +14,+S16,+20	33-54	1
			complex		1
		ICM	N/A		
	**Aneuploid mosaic**				
**27**	B412	TE	2N,XX,**+19**		5
			2N,XX, -S13,**+19**	26-111(~)	2
			complex		2
		ICM	2N,XX,**+19**		7
**<u>28</u>**	B411	TE	2N,XX,**-22**		19
			2N,XX, -S4,**-22**	71-187	1
			complex		1
		ICM	2N,XX,**-22**		1
**29**	B412	TE	2N,XX,**-15**		13
			2N,XX, -S8,**-15**	1-39(~)	4
		ICM	2N,XX,**-15**		3
**<u>30</u>**	B421	TE	2N,XY,**-11**		4
			2N,XY, +18,**-11**		1
		ICM	2N,XY,**-11**		1
**31**	B411	TE	2N,XY,**-13,-18**		11
			2N,XY,-S9,**-13,-18**	70-138	3
			2N,XY, -3,**-13,-18**,+21		1
			2N,XY,+3,**-13,-18**,+21		1
			2N,XY,-3,**-13,-18**		1
			2N,XY,+7,**-13,-18**		1
		ICM	2N,XY, **-13,-18**		2
**32**	B322	TE	2N,XY,**-22**		6
			2N,XY, +S2,**-22**	171-240	1
			2N,XY,-S9,-S17,**-22**	93-126, 6-42	1
			2N,XY,-S5,-S9,**-22**	143-180, 112-138	1
			complex		1
			2N,XY,-S14	47-105	1
		ICM	2N,XY,**-22**		1
**33**	B412	TE	2N,XX,**+4**		3
			2N,XX,+**4**,-S13	65-111	1
			2N,XX,+2,**+4**,-9,-20		1
		ICM	2N,XX,**+4**,-S13	65-111	1
**34**	B411	TE	2N,XY,**-21**		17
			2N,XY,-S20,**-21**	35-64	1
			2N,XY,-17,**-21**		1

		ICM	2N,XY,**-21**		5
			2N,XY,+S20,**-21**	38-64	2
			2N,XY,-S8,**-21**	36-144	1
**35**	B412	TE	2N,XY,**-4**		14
			2N,XY,**-4**,+9		1
			2N,XY,**-4**,+S21	1-22	1
			2N,XY,**-4**,+18		1
			2N,XY,**-4**,-S7	99-159	1
			2N,XY,**-S4,+S4**	1-49, 49-187	1
			4N,XXYY,**-4**,+7		1
		ICM	2N,XY,**-4**		1
			2N,XY**,-4,+**S21	1-22(~)	3
**36**	B412	TE	2N,XY,-4**+9**		3
			2N,XY,-S4,**+9**	164-187(~)	4
			2N,XY,+S4,-S4,**+9**	1-164, 164-187	3
		ICM	2N,XY,-S4,**+9**	159-187	1
			2N,XY,-4+**9**		2
			2N,XY,+S4,-S4,**+9**	1-164, 164-187	1
**41**	B412	TE	**3N**,XXY,+10		9
			**3N**,XXY,-10		4
			**3N**,XXY,+10, +S3	90-197	1
			**3N**,XXY,+10, +S16	64-90	1
			**3N**,XXY,+S10,-S16	1-116, 64-90	1
			**3N**,XXY,+S10	1-100	2
			**6N**,XXY		1
			complex		4
		ICM	**3N**,XXY,-10		1
			**3N**,XXY		1
**42**	B521	TE+ICM	3N,XXY		7
			2N,XY,+9		1
			2N,XY,+S1	150-188	1
			2N,XY,-S1	154-246 (~)	2
			2N,XY,-S1,+18	119-246	1
**43**	B421	TE	3N,XXY		5
			2N,XO,+S8,+S10,+S17	66-144, 100-133(~), 46-83(~)	3
			2N,XX,-22,-SX	1-78	2
			2N,XX,-22,-S1	1-41	1
			3N,XXY,-S22	1-29	1
		ICM	N/A		
**44**	B522	TE	2N,X0		2
			2N,XX,-8		3

			2N,XX,+S9	70-138	1
		ICM	N/A		
	**Uniformly abnormal**				
**37**	B521	TE	N/A		
		ICM	2N,**XO**		5
**38**	B422	TE	2N,XX,**-15**		18
		ICM	N/A		
**39**	B421	TE	2N,XX,**+20**		13
		ICM	N/A		
**40**	B411	TE	2N,XY,**+22**		9
		ICM	2N,XY,**+22**		2
	**Normal**				
**50**	B412	TE	N/A		
		ICM	2N,XY		7
**51**	B422	TE	2N,XY		2
		ICM	2N,XY		1
**52**	B522	TE	2N,XY		3
		ICM	2N,XY		2
**53**	B312	TE	2N,XX		6
		ICM	2N,XX		2
**54**	B422	TE	2N,XY		3
		ICM	2N,XY		1
**55**	B412	TE	2N,XX		3
		ICM	2N,XX		7

Supplemental Table 3: Chromosomal composition of single cells per embryo. For each embryo the morphology score is shown and a distinction is made between cells from the TE and the ICM. Structural abnormalities are presented with an S in front of the involved chromosome. The genomic location with start and end base position (Mb) (NCBI37/hg19) of the segment are also shown. Complex are considered the cells with more than four chromosomal abnormalities Abnormalities in bold most probably originate from meiotic errors. Embryos with an underlined embryo number contain mitotic abnormalities that occurred only in a single cell. TE=Trophectoderm, ICM= Inner cell mass, (~): variation of a few Mb in start or end point of an involved segment in different cells. * Morphological grading according to the Alpha Scientists in Reproductive Medicine and ESHRE Special Interest Group of Embryology consensus on embryo assessment (1).

1. Scientists A, ESHRE Special Interest Group of Embryology. The Istanbul consensus workshop on embryo assessment: proceedings of an expert meeting†. Hum Reprod. 2011;26(6):1270–1283.

Observation	Embryo number	TE + ICM	TE or ICM
Reciprocal loss and gain of whole chromosomes within one embryo (n=8 events)	9,15, 16, 17, 18, 41, 31, 24	15, 16, 17, 18, 41	31 (TE), 24 (ICM), 9 (TE)
Reciprocal loss and gain of chromosomal segments (n=19 events)	19, 20, 25, 23, 24, 22, 21, **42**, **26**, 41, 36, 34	21, 22, 23, 24, 25, 34, 36	19 (TE), 20 (TE), 41 (TE)
Whole and partial loss of the same chromosome (n=8 events)	25, 22, **26**, 3,**11**, **43**, 36	22, 3, 36	25 (TE)
Mixoploidy	42, 43, 44	42	43 (TE), 44 (TE)
Related complex cells	6, **12**, 18, 19, 22, 25, 41	18, 22, 25	6 (TE), 19 (TE), 41 (TE)

Supplemental Table 4: Categorization of observed chromosomal abnormalities. The abnormal cells could be present in both TE and ICM (error event before lineage specification) or in one of the lineages (error event after lineage specification). In bold the embryos where a separate scKaryo-seq result is not available for ICM and TE. According to our definition, complex cells contain more than four abnormalities. The cytogenetic results of the cells for each embryo are shown in Supplemental Table 3. Trophectoderm (TE); Inner cell mass (ICM)

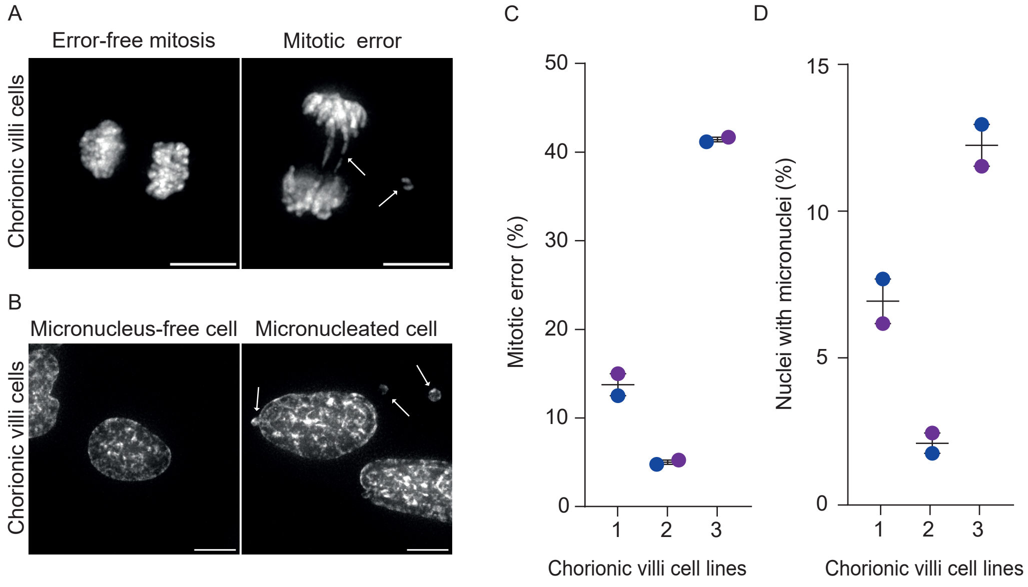

Supplemental Figure 1: Chromosome mis-segregations in chorionic villi cell lines. (A) Representative images of fixed and DAPI-stained chorionic villi cells undergoing error-free mitosis (left image) and with a segregation error (right image). Arrows indicate mis-segregating chromosomes (scale bar = 5 µm). (B) Representative images of chorionic villi cells in interphase without micronuclei (left image) or with micronuclei (right image). Arrows indicate micronuclei (scale bar = 5 µm). (C) Quantification of the mitotic errors in three different chorionic villi cell lines based on the data shown in A. Experiment was performed in duplicate (n = 80 cells in mitosis for the first two cell lines and 29 for the third). (D) Quantification of the micronucleus frequency in three different chorionic villi cell lines based on the data shown in B. Experiment was performed in duplicate (n = 172 interphase cells for the first cell line, 196 for the second and 106 for the third). The blue and purple dots in graph C and D represent the two duplicates.

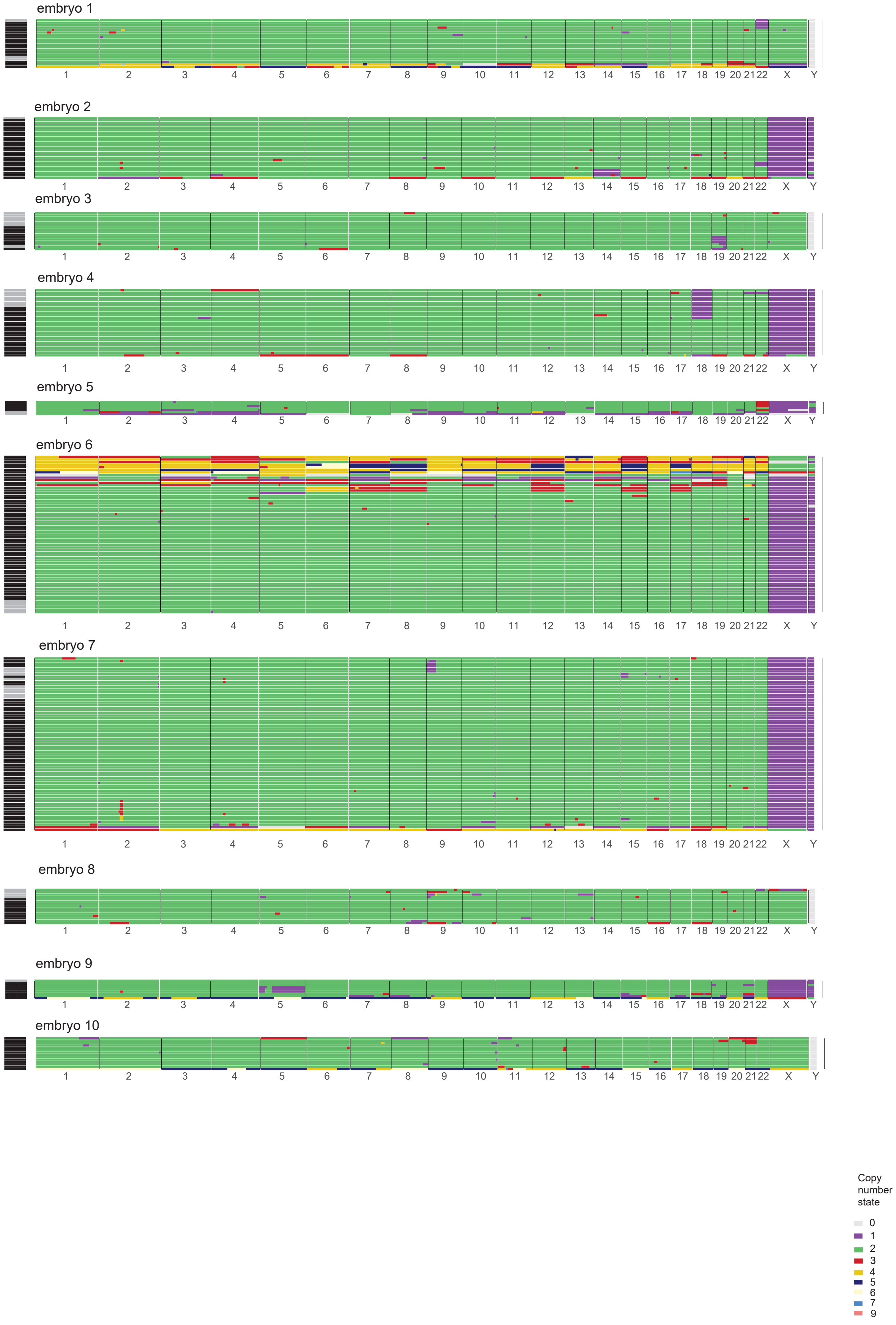
embryo 1
embryo 2
embryo 3
embryo 4
embryo 5
embryo 6
embryo 7
embryo 8
embryo 9
embryo 10
1 2 3 4 5 6 7 8 9 10 11 12 13 14 15 16 17 18 19 20 21 22 X Y
Copy number state
0
1
2
3
4
5
6
7
9

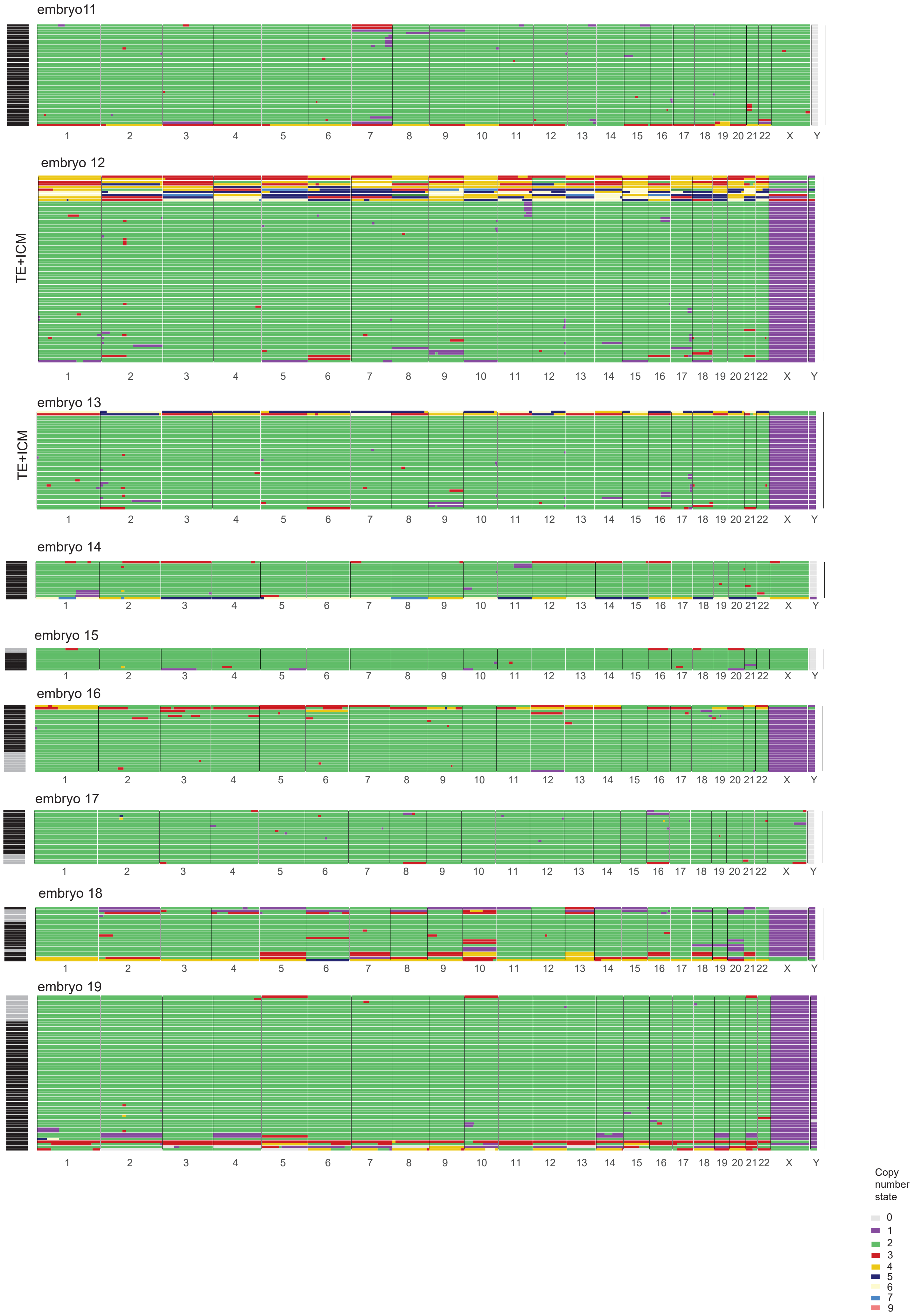

embryo11
embryo 12
TE+ICM
embryo 13
TE+ICM
embryo 14
embryo 15
embryo 16
embryo 17
embryo 18
embryo 19
1 2 3 4 5 6 7 8 9 10 11 12 13 14 15 16 17 18 19 20 21 22 X Y
Copy number state
0
1
2
3
4
5
6
7
9

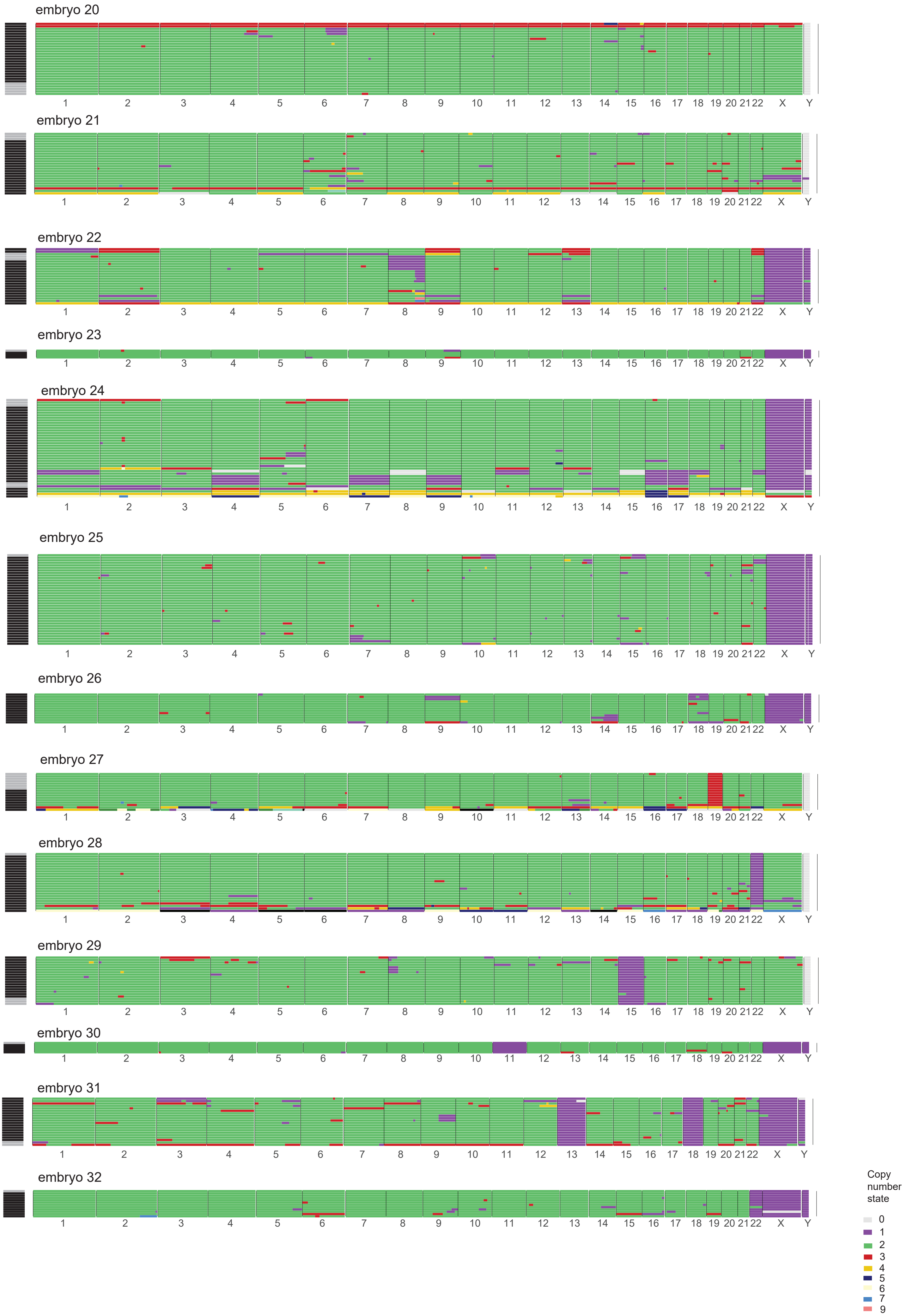
embryo 20
embryo 21
embryo 22
embryo 23
embryo 24
embryo 25
embryo 26
embryo 27
embryo 28
embryo 29
embryo 30
embryo 31
embryo 32
1 2 3 4 5 6 7 8 9 10 11 12 13 14 15 16 17 18 19 20 21 22 X Y
Copy number state
0
1
2
3
4
5
6
7
9

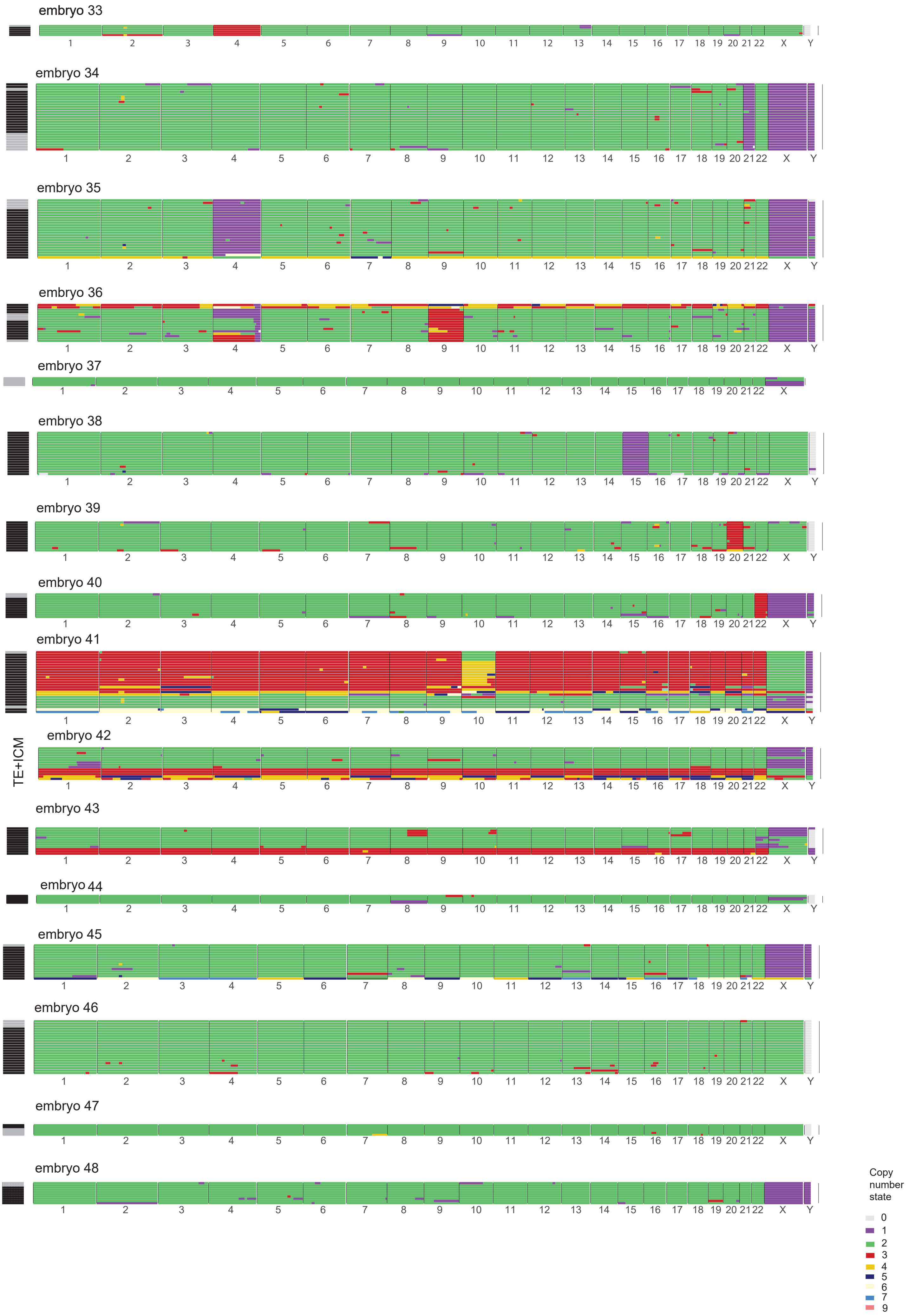
embryo 33
embryo 34
embryo 35
embryo 36
embryo 37
embryo 38
embryo 39
embryo 40
embryo 41
TE+ICM
embryo 42
embryo 43
embryo 44
embryo 45
embryo 46
embryo 47
embryo 48
1 2 3 4 5 6 7 8 9 10 11 12 13 14 15 16 17 18 19 20 21 22 X Y
Copy number state
0
1
2
3
4
5
6
7
9

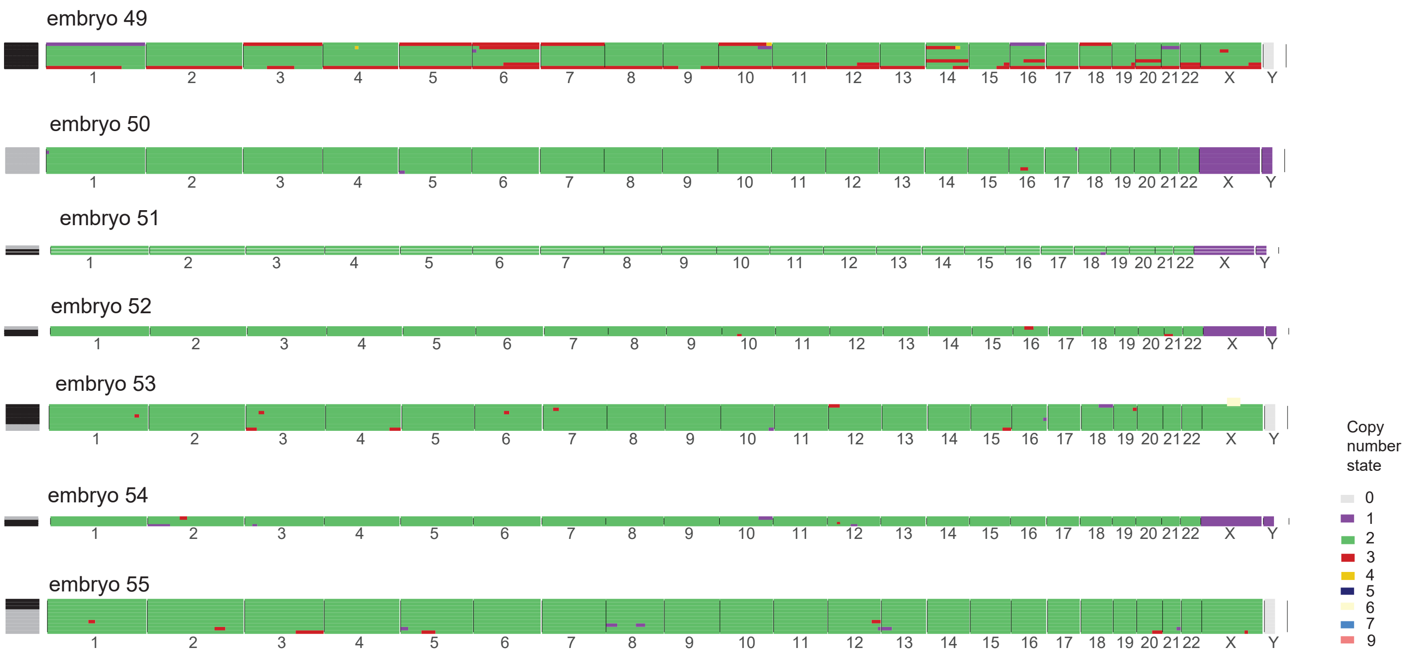

Supplemental Figure 2: scKaryo-seq genome-wide copy number plot for each embryo. For each embryo, all abnormalities per cell are shown regardless of the quality control result. Every row represents a single-cell and every column is a different chromosome. The colors portray copy number states. Colors on the left depict TE (black) or ICM cells (grey). The embryo numbers refer to Supplemental Table 3.
